# Joint ancestry and association test indicate two distinct pathogenic pathways involved in classical dengue fever and dengue shock syndrome

**DOI:** 10.1371/journal.pntd.0006202

**Published:** 2018-02-15

**Authors:** Marisa Oliveira, Worachart Lert-itthiporn, Bruno Cavadas, Verónica Fernandes, Ampaiwan Chuansumrit, Orlando Anunciação, Isabelle Casademont, Fanny Koeth, Marina Penova, Kanchana Tangnararatchakit, Chiea Chuen Khor, Richard Paul, Prida Malasit, Fumihiko Matsuda, Etienne Simon-Lorière, Prapat Suriyaphol, Luisa Pereira, Anavaj Sakuntabhai

**Affiliations:** 1 i3S—Instituto de Investigação e Inovação em Saúde, Universidade do Porto, Porto, Portugal; 2 Instituto de Patologia e Imunologia Molecular da Universidade do Porto (IPATIMUP), Porto, Portugal; 3 Instituto de Ciências Biomédicas Abel Salazar (ICBAS), Universidade do Porto, Porto, Portugal; 4 Functional Genetics of Infectious Diseases Unit, Institut Pasteur, Paris, France; 5 Bioinformatics and Data Management for Research, Office for Research and Development, Faculty of Medicine Siriraj Hospital, Mahidol University, Bangkok, Thailand; 6 Department of Pediatrics, Faculty of Medicine, Ramathibodi Hospital, Mahidol University, Bangkok, Thailand; 7 Pasteur Kyoto International Joint Research Unit for Integrative Vaccinomics, Kyoto, Japan; 8 Center for Genomic Medicine, Kyoto University Graduate School of Medicine, Kyoto, Japan; 9 Genome Institute of Singapore, A-STAR, Singapore, Singapore; 10 Department of Biochemistry, National University of Singapore, Singapore, Singapore; 11 CNRS, Unité de Recherche Associée 3012, Paris, France; 12 Dengue Hemorrhagic Fever Research Unit, Office for Research and Development, Siriraj Hospital, Faculty of Medicine, Mahidol University, Bangkok, Thailand; 13 Medical Biotechnology Unit, National Center for Genetic Engineering and Biotechnology, National Science and Technology Development Agency, Pathumthani, Thailand; 14 Department of Pathology, Faculty of Medicine, University of Porto, Porto, Portugal; Institute for Disease Modeling, UNITED STATES

## Abstract

Ethnic diversity has been long considered as one of the factors explaining why the severe forms of dengue are more prevalent in Southeast Asia than anywhere else. Here we take advantage of the admixed profile of Southeast Asians to perform coupled association-admixture analyses in Thai cohorts. For dengue shock syndrome (DSS), the significant haplotypes are located in genes coding for phospholipase C members (*PLCB4* added to previously reported *PLCE1*), related to inflammation of blood vessels. For dengue fever (DF), we found evidence of significant association with *CHST10*, *AHRR*, *PPP2R5E* and *GRIP1* genes, which participate in the xenobiotic metabolism signaling pathway. We conducted functional analyses for *PPP2R5E*, revealing by immunofluorescence imaging that the coded protein co-localizes with both DENV1 and DENV2 NS5 proteins. Interestingly, only DENV2-NS5 migrated to the nucleus, and a deletion of the predicted top-linking motif in NS5 abolished the nuclear transfer. These observations support the existence of differences between serotypes in their cellular dynamics, which may contribute to differential infection outcome risk. The contribution of the identified genes to the genetic risk render Southeast and Northeast Asian populations more susceptible to both phenotypes, while African populations are best protected against DSS and intermediately protected against DF, and Europeans the best protected against DF but the most susceptible against DSS.

## Author summary

Dengue fever is endemic in tropical and subtropical areas of East Asia and America, but globalization and climate changes are introducing vector and virus to the naïve regions of Europe and North America. In this work we conducted a statistically robust, coupled association-admixture test in two dengue cohorts from Thailand (classical dengue fever, DF, and dengue shock syndrome, DSS) and a published Vietnamese (DSS only) cohort. We identified new candidate genes associated with DF risk and confirmed known gene family association with DSS risk. In DF, phosphatase control is crucial, including through binding to viral proteins, as we showed for PPP2R5E protein co-localization with DENV1 and DENV2-NS5 proteins within liver cells and differential cellular localizations along time. In DSS, cytokine dynamics, inflammation and activation of vascular endothelium cells are dominant features. The particular genetic risk conferred by these genes indicates that Southeast and Northeast Asians are highly susceptible to both phenotypes, while Africans are best protected against DSS, and Europeans best protected against DF but the most susceptible against DSS.

## Introduction

Dengue virus (DENV) is the most common mosquito-borne viral infection, infecting approximately 390 million people per year worldwide with one quarter developing dengue disease (MIM: 614371) [1]. Symptoms range from undifferentiated fever, classical dengue fever (DF) to shock syndrome (DSS; hemorrhage, plasma leakage and vital organ impairment) [2].

Recent *-omic* approaches provide unbiased genomic insights into mechanisms associated with dengue disease. There has been only one publication on classical genome wide association study (GWAS) of dengue [3] compared to a considerable number of transcriptomic studies [4–7]. The reason for this discrepancy is that cohorts of thousands of individuals are required for GWAS to reach genome wide significance. The GWAS work conducted on a cohort of Vietnamese children [3] included 2,008 DSS samples versus 2,018 controls, replicated in 1,737 versus 2,934, and found SNPs in genes *MICB* and *PLCE1* associated with DSS phenotype. Lately, analytical improvements based on admixture mapping have reduced the sample size requirement from thousands to hundreds of individuals or even fewer [8]. Most human populations have some degree of ancestry admixture, which brings together haplotypes that occur at different frequencies in parental populations. Admixture mapping analyses these blocks across the mosaic descendant chromosomes and allows to compare their distribution between case and control cohorts. The lower number of blocks compared with individual SNPs reduces considerably the statistical burden. We have successfully conducted such an admixture study in dengue cohorts from Cuba [9], and identified two genes involved in lipid metabolism which showed to be protective against the risk of dengue hemorrhagic fever, a protection conferred by the African inherited ancestry. Whereas for *RXRA* gene there was already functional evidence of its involvement in infection [10], we also demonstrated functionally by shRNA that the knockdown of *OSBPL10* gene had a significant negative impact in DENV replication rate [9].

Epidemiologic reports have shown the existence of ethnic differences in susceptibility to dengue fever not only in Cuba [11] but also in Malaysia [12] where the incidence rate by ethnic group was 3.7:1:1.3 for Chinese, Malays and Indians, respectively, in the years 1970’s and 1980’s, although no cross-evaluation was performed with other socio-demographic factors. In the present study, we take advantage of the admixed profile of Southeast Asians (in the nexus between South, Northeast and Southeast Asia) to perform coupled association-admixture analyses (BMIX; [13]) of case/control cohorts of dengue patients: Thai dengue patients who developed DF (n = 252) or DSS (n = 159), and a control blood donor group (n = 290); and the published Vietnamese dataset (2018 controls and 2008 DSS patients;[3]). Although the admixture in the region has been taking place along time, since the first arrival of modern human after the out-of-Africa migration, a considerable migration from south China began in the 15th century and increased in the 19th and 20th centuries, mainly towards Thailand where about 40% of the population has some Chinese admixture and 14% are identifiable Thai Chinese [14]. This is a similar scenario to the admixture that took place in the Americas, where these local admixture inference tools have been successfully applied [9, 15, 16]. We were able to identify distinct candidate genes conferring susceptibility/resistance to the risk of DF and DSS, arguing in favor of independent pathogenic mechanisms for the establishment of the two phenotypes. We further confirmed that one DF candidate gene codes for a human protein that co-localizes with the DENV1 and DENV2-NS5 proteins, and, in the latter case, transiently relocated from the cytoplasm to the nucleus. We also inferred the relative worldwide genetic risks contributed by the detected candidate genes based on their frequencies for the susceptible/resistant haplotypes.

## Results

### Ancestry of Thai and Vietnamese cohorts

**Fig 1:**
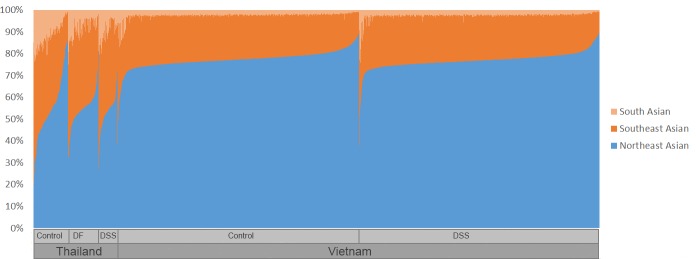
Global ancestry inferred through RFMix when using three parental ancestries (South, Northeast and Southeast Asian) for the global dataset. Each vertical line represents an individual, and the three colours represent the proportion of the three parental populations in each genome (light orange for South Asian, dark orange for Southeast Asian and blue for Northeast Asian).

All analyzed individuals have some degree of admixture (Fig 1; S1 Fig). The Northeast Asian background is dominant in Vietnam (77.3%) and decreases in Thailand (56.4%), in contrast to the Southeast Asian component, which increases from 20.7% in Vietnam to 35.1% in Thailand. The South Asian influence is 8.5% in Thailand and 2.0% in Vietnam. Within the dengue cohorts, we observed a statistically significant increase in the Southeast Asian background in Thailand for both DF (4.1% increase; p-value = 1.25 x 10^−7^) and DSS (4.8% increase; p-value = 5.90 x 10^−8^) compared to Thai control.

### DSS cohorts analyses

**Fig 2:**
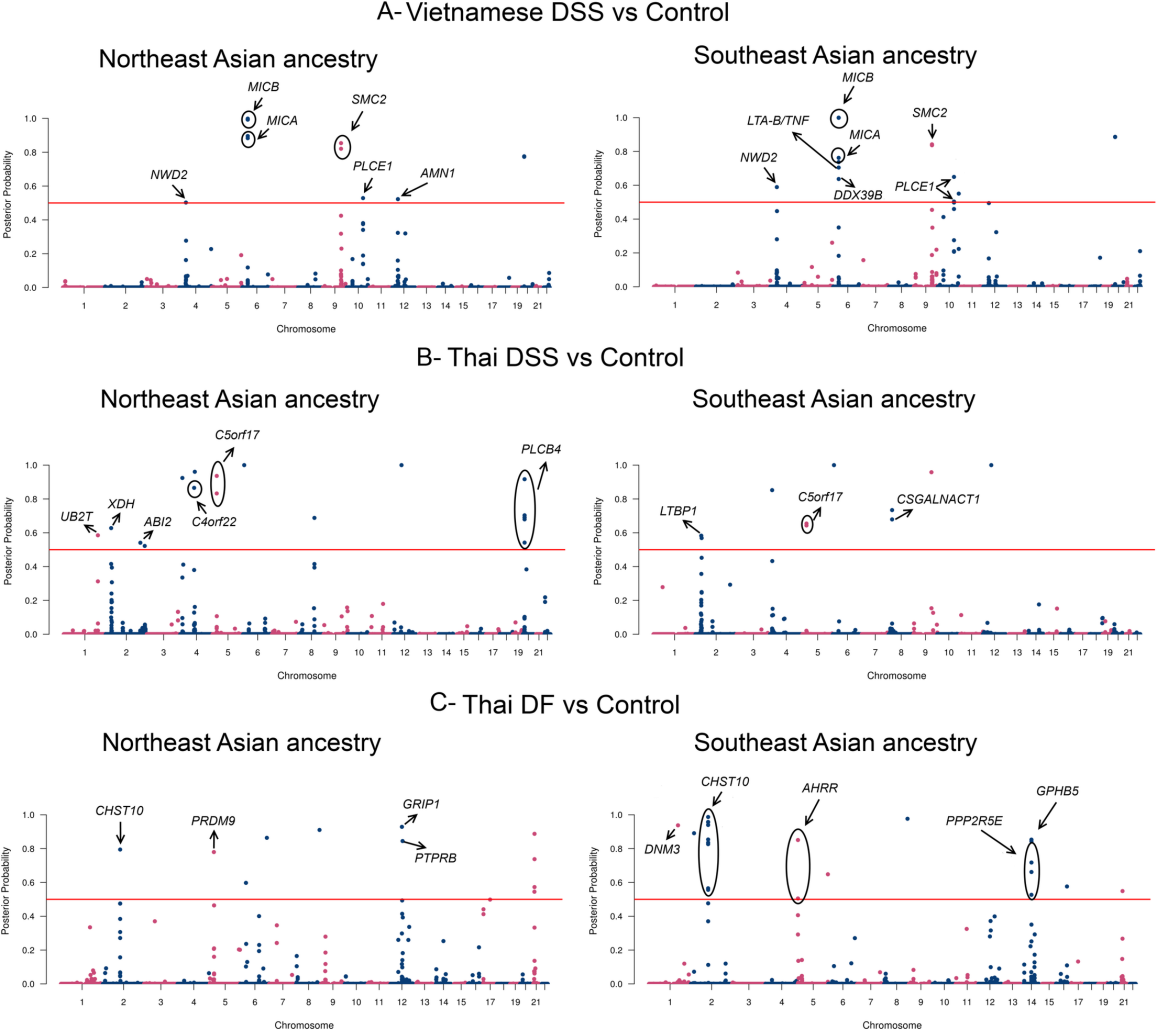
**Manhattan plots of BMIX analysis in Vietnamese DSS vs Control (A), Thai DSS vs Control (B) and Thai DF vs Control (C) for Northeast and Southeast Asian ancestries.** The red line represents the significance threshold. The protein coding genes with significantly associated SNPs are identified.

**Fig 3:**
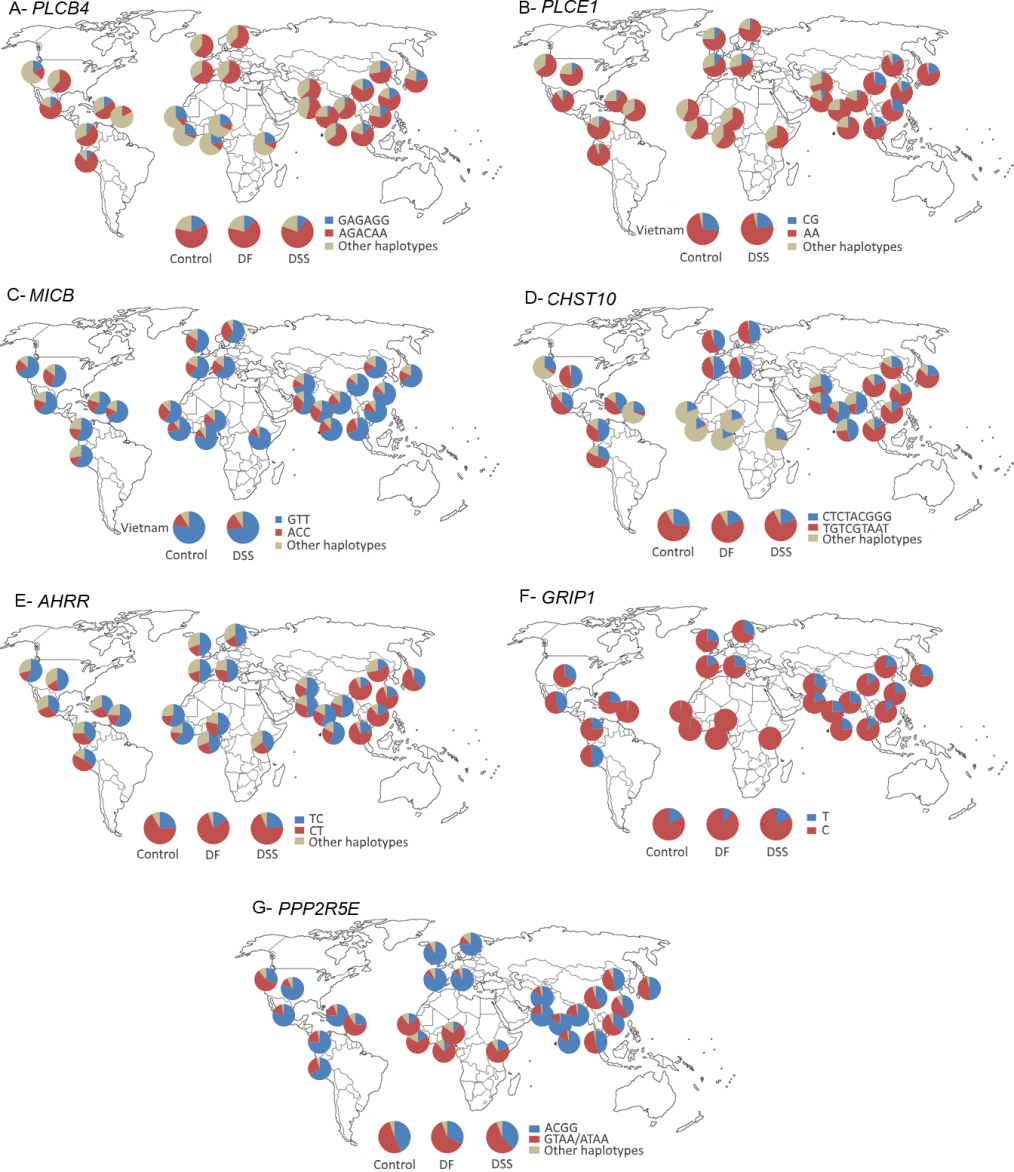
Worldwide (from the 1000 Genomes database) and Thai dengue cohorts (control, DF and DSS) frequencies for significantly associated haplotypes in the various genes. A- *PLCB4*; B- *PLCE1*; C- *MICB*; D- *CHST10*; E- *AHRR*; F- *GRIP1*; G- *PPP2R5E*. The protective and causative haplotypes are highlighted.

We began by checking if the BMIX results on the published Vietnamese cohort [3] are in accordance with the results from the classical association mapping, a test of the robustness of the algorithm. BMIX indicates also the association of DSS with *MICB* and *PLCE1* genes (Table 1, Fig 2A, S1 and S4 Tables). The identified region surrounding *MICB* encompasses seven significant SNPs, placed along 165,080 bp, from the downstream *MICA* to the upstream *LTB* gene, a region highly rich in genes. Three linked (S2 Fig) SNPs in *MICB* have the most significant p-values, forming the protective haplotype GTT (OR = 0.77; p-value<0.0001), which is the most frequent haplotype in worldwide populations (Fig 3C). The susceptible *MICB* haplotype ACC (OR = 1.39; p-value<0.0001) is more frequent in Europeans and South Asians (0.18 to 0.34). The two SNPs found for *PLCE1* reached significant p-values and are almost in complete linkage (S5 Fig). The DSS protective *PLCE1* haplotype (CG; OR = 0.75; p-value<0.0001) is more frequent (Fig 3B) in Northeast Asia (0.12–0.28) and Southeast Asia (0.19), followed by Europe (0.04–0.14) and absent in Africa.

**Table 1:** Odds ratios (ORs), 95% confidence intervals and Yates p-values (corrected for continuity) of the χ2 test for the significant haplotypes/SNPs in the phenotype and populations for which association was detected.

	Protective haplotype				Susceptible haplotype			
Gene	Frequency Control	Frequency Case	OR (95 CI)	P value	Frequency Control	Frequency Case	OR (95 CI)	P value
		Vietnamese DSS study						
*MICB*		rs2534666-rs2855807-rs3132468						
	G-T-T				A-C-C			
	0.78	0.73	0.77 (0.70–0.85)	<1.0 x 10^−4^	0.13	0.18	1.39 (1.23–1.57)	<1.0 x 10^−4^
*PLCE1*		rs3740360-rs2274223						
	C-G				A-A			
	0.27	0.22	0.75 (0.68–0.84)	<1.0 x 10^−4^	0.69	0.75	1.30 (1.18–1.43)	<1.0 x 10^−4^
		Thai DSS study						
*PLCB4*		rs16995800-rs2299676-rs7269910-rs1997696-rs6133707-rs6056595						
	G-A-G-A-G-G				A-G-A-C-A-A			
	0.17	0.10	0.58 (039–0.88)	1.3 x 10^−2^	0.61	0.70	1.48 (1.10–1.98)	1.1 x 10^−2^
		Thai DF study						
*CHST10*		rs4850931-rs1030902-rs2241811-rs2241810-rs4149518-rs2241809-rs4149510-rs4851313-rs3828193						
	C-T-C-T-A-C-G-G-G				T-G-T-C-G-T-A-A-T			
	0.26	0.17	0.59 (0.44–0.79)	5.2 x 10^−4^	0.62	0.74	1.78 (1.37–2.31)	<1.0 x 10^−4^
*AHRR*		rs6555205-rs2721020						
	T-C				C-T			
	0.25	0.16	0.54 (0.40–0.74)	1.2 x 10^−4^	0.67	0.79	1.89 (1.43–2.48)	<1.0 x 10^−4^
*PPP2R5E*		rs3829766-rs6573513-rs743221-rs7144210						
	A-C-G-G				GTAA			
	0.43	0.32	0.62 (0.48–0.79)	2.0 x 10^−4^	0.39	0.50	1.59 (1.25–2.03)	2.0 x 10^−4^
*GRIP1*		rs1480010						
	T				C			
	0.17	0.11	0.56 (0.39–0.80)	1.6 x 10^−3^	0.83	0.89	1.79 (1.26–2.56)	1.6 x 10^−3^

We further analyzed the Thai DSS *vs*. control cohort (Table 1, Fig 2B, S2 and S5 Tables), and obtained a reliable signal of six linked (S3 Fig) significant SNPs for *PLCB4* (phospholipase C, beta 4; S4A Fig), a gene in the same family as *PLCE1*, and participating in many common pathways, such as dendritic cell maturation, PI3K signaling in B lymphocytes and PPARA/RXRA activation. The DSS protective *PLCB4* haplotype (GAGAGG; OR = 0.58; p-value = 0.013) is rare in most worldwide populations (Fig 3A), reaching the highest frequencies in Africa (0.21–0.28). Only one *PLCE1* SNP (rs2274223) was present in the chip used in the Thai dengue cohort and it did not reach significance.

Individually, the conventional association study with PCA correction for population stratification in Thai DSS *vs*. control could not identify any candidate gene when correcting for multiple test (S6A Fig–the two singled out significant SNPs are spurious signals as linked SNPs do not display significant p-values). We also tested 10 runs of pseudo datasets, permutating case and control labels (S1 Text). No SNP is significant in the association tests, and the BMIX algorithm identified spurious significant SNPs (mostly isolated in different chromosomes) that do not replicate between runs and that are different from the case-control comparison. Overall posterior p-values were also lower in the pseudo datasets. The higher spurious detections in BMIX than in the association test agree with the fact that the statistical burden of the local ancestry test is considerably lower than the one for the association test, which raises the possibility of detecting a positive signal. The randomness between runs reflects the high variability between individuals in admixture percentages and in distribution of ancestry blocks along the genomes. This argues for a double-careful interpretation of BMIX results in the context of the disease. For the Thai DSS *vs*. control, the fact that the *PLCB4* gene belongs to the same family of the previously independently identified *PLCE1* gene is an important additional evidence for considering that gene a strong candidate in DSS phenotype.

**Fig 4:**
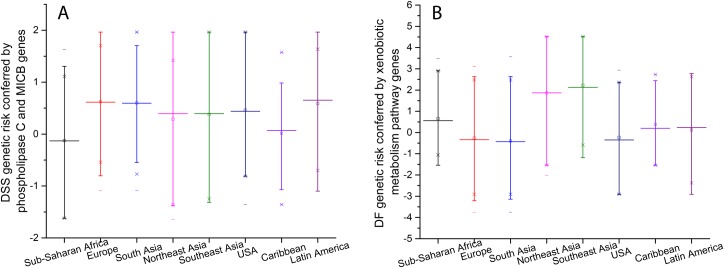
**Genetic risk for the various worldwide regions by considering an additive model of protective and causative haplotypes/SNPs for DSS (A) and DF (B).** Median (middle line), mean (little square), 95% confidence interval (whiskers) and extreme values (crosses) are indicated.

Calculating the genetic risk of DSS according to the worldwide population frequency of the phospholipase C and *MICB* protective and susceptible haplotypes (Fig 4A), it can be observed that African and descendent Caribbean populations are best protected, while European, Asian and Latin American populations are more susceptible to DSS.

We genotyped two of the BMIX-identified significantly associated *PLCB4* SNPs in further Thai control (n = 244) and case (n = 20) samples (S7 Table), and the rs1997696 SNP presents a p-value over the significance threshold of a traditional GWAS (p = 4.7x10^-8^).

### Dengue fever cohort analysis

**Fig 5:**
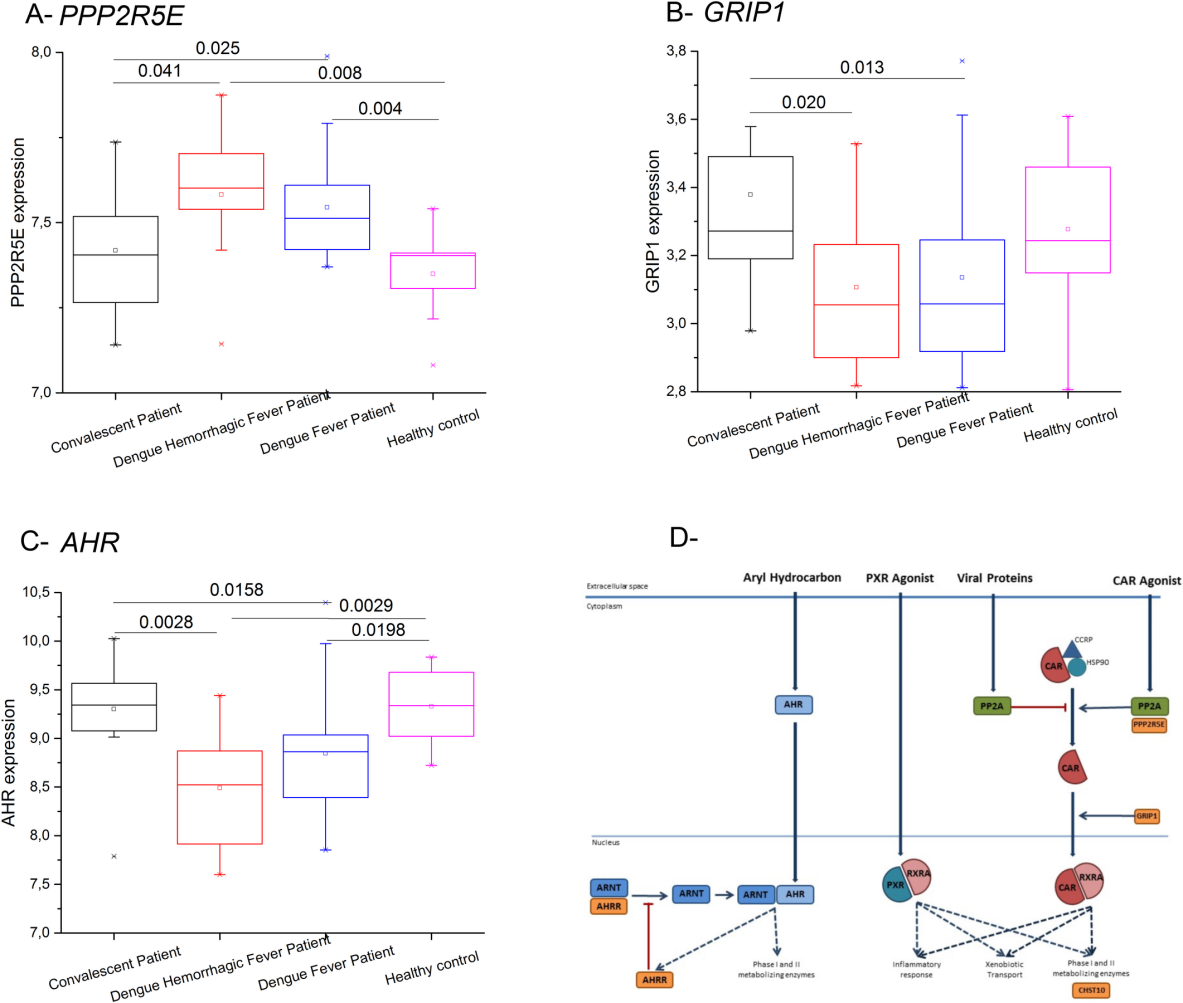
**Significantly altered gene expression for *PPP2R5E* (A), *GRIP1* (B) and *AHR* (C) in Thai dengue cohort along the course of disease from a transcriptome dataset for whole blood[17].** Significant p-values are indicated. D) Scheme of the xenobiotic metabolism signalling pathway (based on Ingenuity database information), highlighting the three nuclear transcription factors: the constitutive active receptor (CAR); the pregnane X receptor (PXR); and the aryl hydrocarbon receptor (AHR).

When comparing Thai DF *vs*. control, a distinctive genetic signature was obtained. Three genes located on different chromosomes had at least two SNPs above the BMIX significant posterior probability threshold of 0.5 (Fig 2C), forming haplotypes (S7, S8 and S9 Figs). *CHST10* codes for carbohydrate sulfotransferase 10 (S4C Fig), has nine significant SNPs (Table 1, S3 and S6 Tables), forming the protective haplotype CTCTACGGG (OR = 0.59; p-value = 0.0005), whereas the haplotype TGTCGTAAT increased risk of DF (OR = 1.78; p-value<0.0001). The protective haplotype is frequent in South Asian populations (0.38–0.57), whereas the susceptible haplotype is frequent in Northeast Asia (0.61–0.74) and very rare in the African populations (Fig 3D). *AHRR* (S4D Fig) codes for aryl-hydrocarbon receptor (*AHR*) repressor, has two significant SNPs, and similarly to *CHST10*, the protective *AHRR* haplotype (TC—OR = 0.54; p-value = 0.0001) is more frequent in South Asian and African populations (between 0.40–0.60) and the opposite haplotype (CT—OR = 1.89; p-value<0.0001) is more frequent in Northeast Asian populations (0.57–0.78) (Fig 3E). *PPP2R5E* (S4F Fig) codes for protein phosphatase 2 (PP2A), regulatory subunit B', epsilon isoform (also known as PP2A-B56), has four significant SNPs, whose protective haplotype (ACGG—OR = 0.62; p-value = 0.0002) showed high frequency in South Asian populations (0.76–0.86), while African populations have the lowest frequency of this haplotype (0.09–0.20) (Fig 3G). Interestingly, the proteins coded by these three genes, and by another gene, *GRIP1* (S4E Fig) that codes for glutamate receptor interacting protein 1, with one significant SNP (T; OR = 0.56; p-value = 0.0016), are involved in the xenobiotic metabolism signaling pathway (Fig 5D). The *GRIP1* protective allele is more frequent in South Asian populations (0.19–0.32) and absent in Africa (Fig 3F).

Again, individually, the conventional association study with PCA correction for population stratification could not identify any candidate gene when correcting for multiple test (S6B Fig).

The contribution to the genetic risk to DF, inferred from an additive model combining the protective and susceptible haplotypes of the four xenobiotic-related genes (Fig 4B), indicates highest protection in European and South Asian populations and highest risk in Northeast and Southeast Asians. African and Latin American/Caribbean populations have an intermediate risk conferred by these genes to DF.

The genotyping of six SNPs in these four genes in additional Thai controls (n = 245) and cases (n = 55) improves p-values of a traditional association test in the total cohort to levels of 10^*−*5^ in four SNPs and 10^*−*4^ in two SNPs (S7 Table). These values are significant after Bonferroni correction for the set-test of six SNPs.

We analyzed the expression of these genes in the xenobiotic pathway in a transcriptome dataset including patients sampled during acute phase of DF, DHF and convalescence compared with controls [17]. *CHST10* and *AHRR* expressions did not significantly change during dengue infection (S10 Fig), however, there was a significant increase in *PPP2R5E* expression and a significant decrease in *AHR* (negatively regulated by *AHRR*) and *GRIP1* expressions during acute dengue infection (Fig 5A, 5B and 5C). These findings are further evidence that *PPP2R5E*, *GRIP1* and *AHR* can be involved in dengue infection and development of dengue disease. We further checked in the GTEx database if the DF candidate SNPs act as eQTLs. All candidate protective alleles in *PPP2R5E* and *AHRR* genes significantly reduce the expression of the respective proteins (S11 Fig). The candidate SNP in *GRIP1* gene is not an eQTL in the GTEx cohort, and the two eQTLs (rs11176317 and rs12322014) close to the candidate rs1480010 are not in LD with it. As the GTEx cohort is mainly of European ancestry, we cannot ascertain if this *GRIP1* SNP or other linked SNPs can be eQTLs in Asian populations.

### Immunofluorescence co-localization imaging of PPP2R5E and NS5 protein from DENV1 and 2

**Fig 6:**
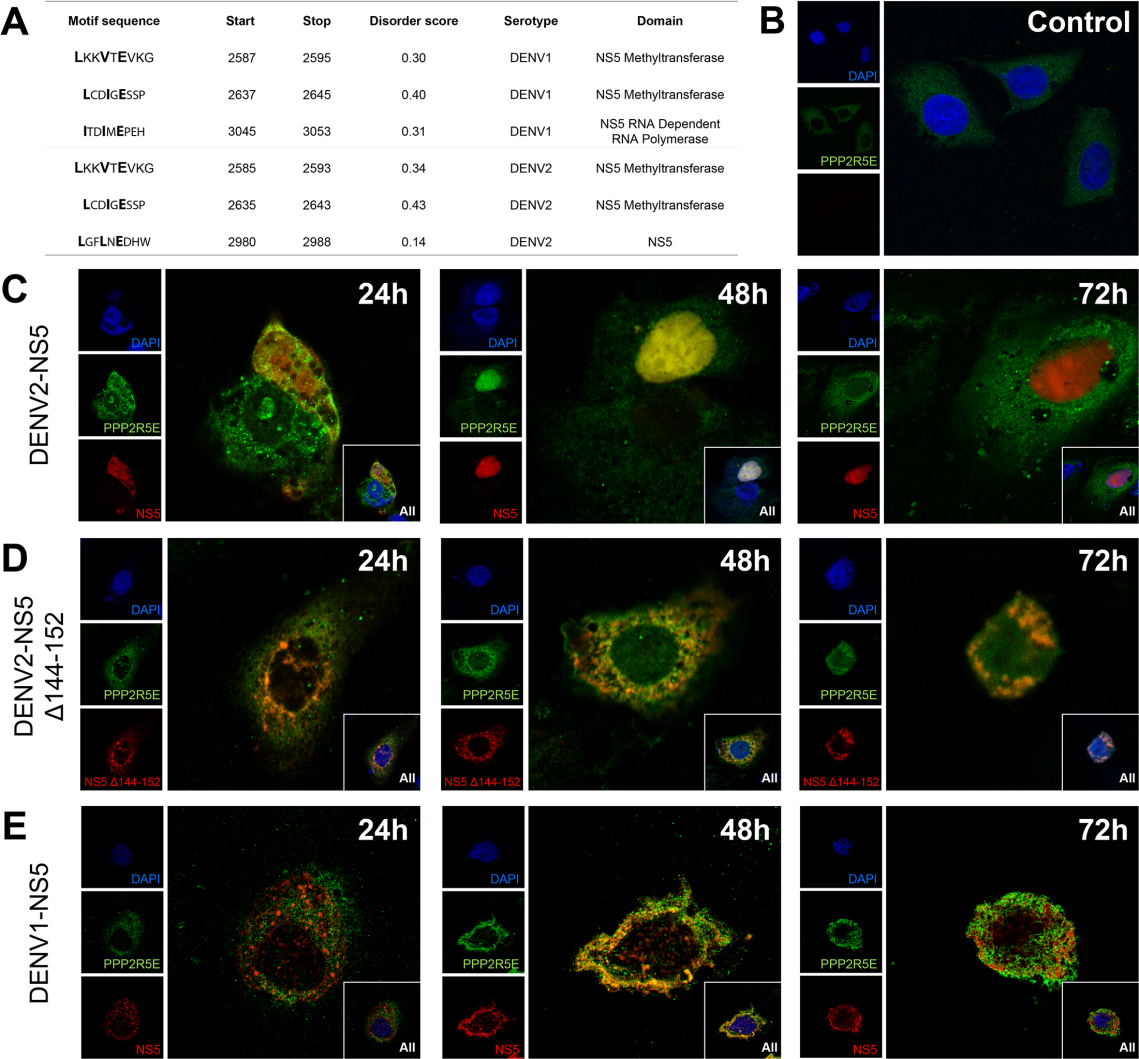
Confocal imaging of PPP2R5E and NS5 from DENV1 and DENV2. A. The main link motifs in NS5 proteins from DENV1 and DENV2. B. Subcellular localization of PPP2R5E in Huh7 control cells. C. Subcellular localization of PPP2R5E in Huh7 control and after 24h, 48h and 72h of transfection with DENV2-NS5 protein. D. Subcellular localization of mutated PPP2R5E in Huh7 control and after 24h, 48h and 72h of transfection with DENV2-NS5 protein. E. Subcellular localization of PPP2R5E in Huh7 control and after 24h, 48h and 72h of transfection with DENV1-NS5 protein. Green immunofluorescence indicates PPP2R5E, red indicates NS5, blue flags nucleus. Yellow signals indicate co-localization of NS5 and PPP2R5E, and was obtained by overlapping the two panels. Original magnification, ×630.

The recent identification of conserved motifs that provide binding specificity to the PP2A-B56 phosphatase [18] led us to further test the hypothesis of the potential binding of this regulatory region of PP2A protein to DENV proteins. We began by performing an *in silico* search [19] for the high-affinity LxxIxE motif as well as the intermediate- and low-affinity motifs in the protein reference sequences of the four DENV serotypes (S8 Table and Fig 6A). NS5 presents between three and six motifs in all four DENV serotypes, and at least two of these motifs (LxxIxE and LxxVxE) are highly conserved. Other viral proteins also bear motifs, but are more heterogeneous between DENV serotypes.

We then tested the hypothesis that PP2A-B56 can interact with NS5 by conducting confocal immunofluorescence co-localization tests. We transfected Huh7 cells with a mammalian expression plasmid containing DENV2-NS5 tagged with an orange fluorescence protein. We fixed and stained with antibody against PPP2R5E at 24h, 48h and 72h after transfection. In non-transfected cells, PPP2R5E is localized in the cytoplasm (Fig 6B). At 24h of post-transfection, both PPP2R5E and NS5 are localized in the cytoplasm, but by 48h they both co-localize in the nucleus, and at 72h PPP2R5E returns to the cytoplasm while NS5 remains in the nucleus (Fig 6C). We then deleted the xLxxIxE motif in our DENV2-NS5 vector (Fig 6D) and transfected cells in the same way. The deletion of this motif prevented the translocation of the viral NS5 protein to the nucleus (Fig 6D).

Testifying to the existence of differences between serotypes, the immunofluorescence co-localization test between PPP2R5E and DENV1-NS5 (Fig 6E) showed that the two proteins co-localize in the cytoplasm, but the entrance in the nucleus is almost negligible, and little accumulation of NS5 can be detected in the nucleus at 72h.

## Discussion

Our successful association-admixture analyses in Thai population have provided evidence that different genes/pathways contribute to the genetic susceptibility or resistance to different outcome of dengue infection. We suggest that xenobiotics and lipid metabolism, as well as interaction of viral proteins to these molecules and to its phosphatases, are critical in the development of classical DF, whereas more severe forms of dengue are caused by over reactive immunity leading to cytokine storm and/or defect in endothelial cell dysfunction and coagulation system.

We reinforced the association of phospholipase C gene family with the DSS phenotype in Thai patients, as had been found in Vietnamese DSS patients [3], but this time the gene detected was *PLCB4* instead of *PLCE1*. These enzymes have been implicated in a high number of signal transduction pathways [19] and on immune regulation [20]. More recently, Lin et al. [21] found *PLCB4*/*PLCB1* susceptibility loci for coronary artery aneurysm in Kawasaki disease when analyzing a Han Chinese cohort. During the acute stage of this disease, inflammation occurs by the infiltration of T cells and macrophages and the activation of vascular endothelium cells (ECs) with increased serum proinflammatory cytokines and predominant damage of small-, medium-sized vessels and the coronary artery. The injured vascular tissues show subendothelial edema, vascular damage, gap formation, and fenestration of ECs. It thus seems that phospholipase C genes are involved in several diseases presenting the phenotype of inflammation of the blood vessels, as it is the case in dengue shock syndrome.

The association of the four genes (*CHST10*, *AHRR*, *PPP2R5E* and *GRIP1*) from the xenobiotic metabolism signaling pathway with Thai DF patients is the first genetic evidence for its implication in dengue pathogenesis, although functional studies have already indicated this association before. Xenobiotics are toxic non-endogenous compounds that together with other toxic endogenous compounds must be eliminated from the body by drug/xenobiotic metabolizing enzymes (DME/XME) and transporters. The DMEs are induced by their own substrates, through signaling cascades involving three specific receptors: the constitutive active receptor (CAR), the pregnane X receptor (PXR) and the aryl hydrocarbon receptor (AHR) (Fig 5D) [22]. In particular PP2A regulates the CAR:HSP90 complex, allowing CAR release and its eventual translocation to the nucleus [23], an event that could be similar/parallel to PP2A involvement in the nuclear translocation of viral proteins. Several lines of evidence show that some of PP2A regulatory (such as PP2A-B56) and scaffold subunits can bind viral proteins [24–26]. In addition, the capsid of West Nile virus, a flavivirus related to DENV, binds to the inhibitor of PP2A proteins (I_2_^PP2A^ or SET) in the precise site where I_2_^PP2A^ binds to PP2A, causing an increase of PP2A activity in several cell types [27]. These evidences led us to perform immunofluorescence assays that revealed that PPP2R5E co-localizes with the NS5 protein of DENV2, first in the cytoplasm and then in the nucleus, and that this nuclear translocation does not take place when the specific link motif is deleted from the NS5 protein. NS5 is a crucial viral protein responsible for the virus replication at the endoplasmic reticulum. NS5 accumulation in the nucleus seems to occur late in DENV infection as a hyperphosphorylated form unable to bind NS3. Adding a layer of complexity to these events, the test of the NS5 nuclear translocation in the four DENV serotypes showed that it only occurs in DENV2 and DENV3 [28], leading the authors to hypothesize that the NS5 nuclear localization is not strictly required for virus replication but that it is more likely to have an auxiliary function in the life cycle of specific DENV serotypes. Our *in silico* analysis indicates that all NS5 proteins from the four DENV strains contain the specific link-motifs, but our immunofluorescence assay with DENV1-NS5 confirmed the observation of the authors, that the protein of this serotype does not enter the nucleus. Extra factors, such as importin proteins, must contribute to this differential import into the nucleus. Thus, the dynamics of NS5 seems to play a major role in dengue infection, potentially impacting differential strain virulence, and the PPP2R5E-NS5 interaction must be taken into consideration in future studies.

Increased PP2A activity could favor viral infection not only through binding of viral proteins but also through regulation of regulatory T cells (Treg) [29]. Suppression and impairment of anti-viral activity of interferon α (IFNα) has been shown in hepatitis C infection through inhibition of Jak1/Tyk2/STAT1 phosphorylation [30] and upregulation of PP2A dependent upon NS5A protein [24]. Both Treg and Jak1/Tyk2/STAT1 pathway have been shown to be important in DENV infection [31–34].

The significant association detected for *AHRR* and lower expression of AhR during acute dengue infection suggests another possible key factor in dengue infection outcome. AhR has been shown to be involved in mediating the biotransformation and carcinogenic/teratogenic effects of environmental toxins. Recently, its role in innate and adaptive immunity has been demonstrated, through its involvement in regulation of CD4, CD8 and Treg after viral infection [35]. We hypothesize that the outcome of dengue infection depends on a fine-tuning of the xenobiotic metabolism signaling pathway between the AhR and PP2A/CAR pathway. While increased PP2A/CAR activity promotes viral infection, decreased AhR activity results in uncontrolled immune homeostasis leading to dengue disease. The confirmation that our candidate protective alleles in *PPP2R5E* and *AHRR* genes are eQTLs leading to lower expression of the respective proteins supports this hypothesis. When cells are invaded by DENV, there will be an increase in the expression of *PPP2R5E*, possibly due to the interaction with NS5. So Asian individuals that are genetically *PPP2R5E*-low-expressing are protected against this link. For the *AHRR* gene, the protective phenotype also has a lower expression profile, and as this protein will inhibit AHR protein, these individuals will have a higher expression of AHR, which is opposite to the expression pattern observed in the dengue patients transcriptome.

For both DF and DSS phenotypes, Northeast and Southeast Asian populations have a higher ancestral prone risk when compared with other geographical regions, considering the particular genes identified in this work. Specifically, Southeast Asian ancestry has a slightly higher risk for DF than Northeast Asian ancestry (Fig 4B). These genetic predictions agree with observations that almost 75% of the global population exposed to dengue live in Asia-Pacific, with rates of severe dengue being 18 times higher in this region compared with the Americas [36]. African and its descendant populations are the most protected ones against DSS, and displaying an intermediate protection against DF, adding genetic evidence to previous claims that this ancestry is protected against worse dengue phenotypes [37, 38]. Our inferred genetic risk for DF in Africa, slightly higher that the risk in America, agrees quite well with the risk predictions inferred by Bhatt et al. [1] of 16% and 14%, respectively, of the global burden. Climatic change and globalization are enlarging the spread of dengue vector and virus to northern latitudes, putting Europe and North America at risk of autochthonous infections [39]. The considerable number of autochthonous infections that occurred in Madeira Island, Portugal, in 2012/2013 [40] is the first example of a reality that can take place in a near future in continental Europe. The genetic risk calculated here, for the newly and confirmed susceptible/resistant haplotypes, shows that European populations (as well as South Asian and USA) present an even higher risk than Southeast Asian populations to DSS, while they are the best protected ones against DF.

## Materials and methods

### Samples and genotyping

We enrolled 411 patients (age range, 1–31 years; male to female ratio, 0.984) with symptomatic DENV infection during 2000–2003 from two hospitals in Bangkok (Ramathibodi, and Siriraj) and one in Khonkaen, Thailand (S1 Fig). These patients were first admitted to the hospitals with suspicions of dengue infection based on clinical features, and DENV infection was later confirmed by either detection of viral genome or a comparable immunoglobulin G (IgG) and immunoglobulin M (IgM) titers, measured by an enzyme-linked immunosorbent assay, in late acute and/or convalescent sera. Dengue severity was defined according to 1997 World Health Organization (WHO) criteria [2], and we ended up with the following case cohorts: 252 patients with DF with no evidence of plasma leakage but incapacitating dengue fever; 159 with severe plasma leakage and/or bleeding, leading to shock or profound shock (grades 3 and 4). Information about the DENV serotype and primary/secondary infection are provided in S9 Table. These case samples were genotyped with the Illumina Human 660W Quad BeadChip (Illumina, San Diego, CA, USA). The control cohort was collected in the same hospitals, geographically matching the cases, consisting in 290 healthy individuals with no fever and being treated for minor injuries. Control individuals were genotyped with the Illumina HumanOmniExpress BeadChip.

Quality control was performed in PLINK [41], and SNPs with more than 5% missing genotypes, minor allele frequency (MAF) below 5%, and Hardy-Weinberg equilibrium (HWE) deviation p-values of less than 0.001 were filtered out from downstream analyses. We also checked visually for outliers in principal-component analysis (PCA; S12 Fig), and excluded samples that had evidence of being a second-degree relative or closer to another sample in the study (identity by descent >30%; or identity by state >90%). All studied samples passed these criteria. SNPs located in X and Y chromosomes and in mitochondrial DNA were removed from the analyses, leading to a final account of common 261,660 autosomal SNPs.

The Vietnamese cohort consisted in 2018 controls and 2008 DSS patients typed with the Illumina Human 660W Quad BeadChip, amounting in 479,905 SNPs after QC.

Eight SNPs detected in the BMIX analyses were genotyped in additional Thai cohort samples (61 DF; 20 DSS; and 250 controls), through TaqMan assays (Life Technologies, Carlsbad, CA, USA) with commercial probes for the SNPs. The screen was conducted on a QuantStudio 12K Flex (Life Technologies, Carlsbad, CA, USA) and the results were analyzed in TaqManGenotyper software (Life Technologies, Carlsbad, CA, USA).

### Ethics statement

Written informed consent was obtained from all subjects or, in case of individuals under 18 years of age, from their parents or tutors. The protocol was approved by the ethics committees from the Faculty of Medicine, Ramathibodi Hospital, Mahidol University; the Faculty of Medicine, Siriraj Hospital, Mahidol University; the Khon Kaen Hospital; and the Thailand Ministry of Public Health.

### Association test, admixture mapping and BMIX

The tests were conducted in the following comparisons: Thai DF versus controls (DF test); Thai DSS versus controls (DSS test); and Vietnamese DSS versus controls (DSS test). Samples were phased in SHAPEIT v.2 [42] using HapMap reference panel and fine-scale genetic map.

For admixture mapping, we applied the RFMix algorithm [43] and used three ancestral data sets: the phased data from the 1,000 Genomes Database [44] for the Chinese Dai in Xishuangbanna (CDX); and the Indian Telugu from the UK (ITU) representing the Northeast and South Asian ancestries, respectively; and the Malaysian complete genomes from Singapore [45] representing the Southeast Asian ancestry. We checked if the global admixture profile inferred by RFMix would be reproduced when using ADMIXTURE analysis [46] for K = 3 running together the parental populations and Thai and Vietnamese cohorts. As can be verified in S13 Fig, the three-background admixture profiles for the Thai and Vietnamese individuals are identical between RFMix and ADMIXTURE analysis. ITU population is quite homogeneous, while CDX and Malaysians have themselves around 20–30% admixture, but this does not affect the overall proportion inference of the three components. Information on the three ancestry backgrounds was obtained for each locus along chromosomes for every individual, and these values were averaged in each cohort. Two-tailed Mann-Whitney test (non-parametric test, not requiring normal distribution) was applied to assess the significance between the global ancestry proportions inferred for the three ancestral backgrounds within the Thai and Vietnamese cohorts.

BMIX [13] was implemented on the Thai and Vietnamese groups, by coupling the admixture mapping inferred through the RFMix algorithm with the association data. SNPs with a posterior probability of a joint ancestry and association effect equal or higher to 0.5 were considered significant. The annotation of the significant SNPs was inferred by using the Variant Effect Predictor (VEP) tool from Ensembl. The involvement of the significant genes in pathways was checked in the Ingenuity Pathway Database (https://targetexplorer.ingenuity.com/index.htm).

### Calculation of genetic risk

Odds ratios (ORs), 95% confidence intervals, beta parameters and Yates p-values (corrected for continuity) for the significant haplotypes/SNPs in DF and DSS phenotypes were calculated in http://vassarstats.net/odds2x2.html. The dengue phenotype (DF or DSS) genetic risk score was calculated by multiplying each individual’s significant haplotype/allele (either protective or causative) count for each locus by the respective beta coefficient and summing the product for all loci (as in [47]). For DF, the four genes (*CHST10*, *AHRR*, *PPP2R5E* and *GRIP1*) participating in the xenobiotic metabolism signaling pathway were considered. For DSS, both phospholipase C (*PLCB4* and *PLCE1*) and *MICB* genes were included in the calculation.

### Expression data

Expression of candidate genes was checked in a Thai whole blood transcriptome cohort [17] measured with the Human U133 Plus 2.0 Arrays (Affymetrix, Santa Clara, CA, USA), including: nine healthy controls samples; 28 samples collected between days 2 and 9 after onset of symptoms (acute illness) from secondarily infected patients (18 DF and 10 DHF); and 19 samples collected at convalescence, four weeks or later after discharge.

### Cell culture, transfection and co-localization assay

Huh7 liver cell line was cultured in DMEM (Life Technologies, Carlsbad, CA, USA) with 10% bovine serum and penicillin-streptomycin and maintained at 37°C in 5% CO2 and used for three transfection assays: wild type DENV2-NS5; LxxIxE-deleted DENV2-NS5; and wild type DENV1-NS5. The DENV2-NS5 was fused with an orange fluorescent protein and cloned into pCMV3-C-His vector (DENV-2 (strain New Guinea C–GenBank: AF038403) NS5 open reading frame (ORF) (2700bp) mammalian expression plasmid; SinoBiological, Beijing, China). The wild-type DENV2-NS5 clones were mutagenized with the Q5 Site-Directed Mutagenesis Kit (New England Biolabs, Ipswich, MA, USA), following the manufacturers’ protocol. DENV1-NS5 (isolate KDH0026A, Kamphaeng Phet Provincial Hospital, Thailand–GenBank: HG316481) was also fused with OFP and cloned into the same vector using overlapping primers and the HiFi DNA Assembly Protocol (New England Biolabs, Ipswich, MA, USA), according to the manufacturers’ recommendations. The designed primers are described on S10 Table, and the LxxIxE-deletion and DENV1-NS5 assembly were confirmed by Sanger sequencing performed in a 3130xl Genetic Analyzer (Applied Biosystems, Foster City, CA, USA).

Transient transfections were performed using Lipofectamine 3000 reagent (Invitrogen) according to the manufacturer’s protocol. Wildtype and mutated DENV2-NS5 proteins expressions were confirmed in ZOE Fluorescent Cell Imager (Bio-Rad, Hercules, CA, USA). Transfected cells were harvested 24h, 48h and 72h after transfection and fixed with 4% paraformaldehyde. PPP2R5E was tagged with a primary rabbit anti-PPP2R5E antibody (Atlas Antibodies, Bromma, Sweden) and revealed with a secondary goat anti-rabbit Alexa Fluor 488 antibody (Thermo Fisher Scientific, Waltham, MA, USA). Imaging was obtained on a TCS SP5 II (Leica, Wetzlar, Germany) Laser Scanning Confocal microscope. Images (green and red channel) were aligned with Huygens Software (Chromatic Aberration Corrector module), by using 0.2 μm TetraSpeck Microspheres (Thermo Fisher Scientific, Waltham, MA, USA) embedded in the same conditions as the samples. The images were merged and adjusted for brightness and contrast with Fiji software [48].

## Supporting information

S1 Fig Global ancestry inferred through RFMix when using three parental ancestries (South, Northeast and Southeast Asian) for the three Thai hospital dengue cohorts. Each vertical line represents an individual, and the three colours represent the proportion of the three parental populations in each genome (light orange for South Asian, dark orange for Southeast Asian and blue for Northeast Asian).
(DOCX)

S2 Fig LD (D’) values for the *MICB* region in the Chinese population (CDX) from 1000 Genomes database. BMIX identified significant SNPs are indicated by a box. All SNPs have at least 5% minimum allele frequency in the population analysed.
(DOCX)

S3 Fig LD (D’) values for the *PLCB4* region in the Chinese population (CDX) from 1000 Genomes database. BMIX identified significant SNPs are indicated by a box. All SNPs have at least 5% minimum allele frequency in the population analysed.
(DOCX)

S4 Fig **Locus zoom of the chromosomal region around genes (A–*PLCB4*, B–*PLCE1*, C–*CHST10*, D–*AHRR*, E–*GRIP1*, F–*PPP2R5E*) with significant p-values obtained for DSS and DF tests.** The Asian recombination map was used.
(DOCX)

S5 Fig LD (D’) values for the *PLCE1* region in the Chinese population (CDX) from 1000 Genomes database. BMIX identified significant SNPs are indicated by a box. All SNPs have at least 5% minimum allele frequency in the population analysed.
(DOCX)

S6 Fig Manhattan plots for the conventional association tests with PCA correction for population structure. A–DSS test (p-values and D’ for SNPs surrounding the two spurious SNPs are highlighted). B—DF test. The red line indicates the significance threshold.
(DOCX)

S7 Fig LD (D’) values for the *CHST10* region in the Chinese population (CDX) from 1000 Genomes database. BMIX identified significant SNPs are indicated by a box. All SNPs have at least 5% minimum allele frequency in the population analysed.
(DOCX)

S8 Fig LD (D’) values for the *AHRR* region in the Chinese population (CDX) from 1000 Genomes database. BMIX identified significant SNPs are indicated by a box. All SNPs have at least 5% minimum allele frequency in the population analysed.
(DOCX)

S9 Fig LD (D’) values for the *PPP2R5E* region in the Chinese population (CDX) from 1000 Genomes database. BMIX identified significant SNPs are indicated by a box. All SNPs have at least 5% minimum allele frequency in the population analysed.
(DOCX)

S10 Fig Gene expression for *CHST10* (A) and *AHRR* (B) in Thai dengue cohort along the course of disease from a transcriptome dataset for whole blood.[17] No significant differences in expression were observed.
(DOCX)

S11 Fig mRNA expression profiles for the eQTLs in *PPP2R5E* and *AHRR* genes (information from GTEx database). The protective alleles are indicated in green while the causative alleles are in red.
(DOCX)

S12 Fig PCA of the Thai samples. Plot of PC1 versus PC2 in the Thai control and patient cohorts.
(DOCX)

S13 Fig ADMIXTURE plot for K = 3 for Thai and Vietnamese cohorts and the parental populations used in this work (CDX–Chinese Dai in Xishuangbanna; ITU–Indian Telugu from the UK (ITU); and MAL–Malaysian). (DOCX)

S1 Table Significant SNPs in BMIX analysis for Vietnam DSS test. The base position refers to GRCh37 genome assembly.
(DOCX)

S2 Table Significant SNPs in BMIX analysis for Thai DSS vs control test. The base position refers to GRCh37 genome assembly.
(DOCX)

S3 Table Significant SNPs in BMIX analysis for Thai DF test. The base position refers to GRCh37 genome assembly.
(DOCX)

S4 Table Annotation of the significant SNPs in BMIX analysis for Vietnam DSS test, inferred by using the Variant Effect Predictor (VEP) tool from Ensemble. (DOCX)

S5 Table Annotation of the significant SNPs in BMIX analysis for Thai DSS test, inferred by using the Variant Effect Predictor (VEP) tool from Ensemble. (DOCX)

S6 Table Annotation of the significant SNPs in BMIX analysis for Thai DF test, inferred by using the Variant Effect Predictor (VEP) tool from Ensemble. (DOCX)

S7 Table Association p-values in the entire Thai cohort in six and two SNPs selected from the sets of BMIX-associated SNPs with DF and DSS phenotypes, respectively. (DOCX)

S8 Table Detailed identification of motif sequences across DENV serotypes. (DOCX)

S9 Table Information on DENV serotype and primary/secondary in the Thai cohort. (DOCX)

S10 Table Primers used for mutagenesis and DNA assembly protocols. (DOCX)

S1 Text Association and BMIX results for the 10 runs of pseudo datasets, permutating case and control labels. (DOCX)
