# Supplementary material for: Joint ancestry and association test indicate two distinct pathogenic pathways involved in classical dengue fever and dengue shock syndrome
Source: PLoS Negl Trop Dis. 2018 Feb 15;12(2):e0006202. doi: 10.1371/journal.pntd.0006202 (PMC5813895; doi:10.1371/journal.pntd.0006202)
Supplement: S1 Fig — Each vertical line represents an individual, and the three colours represent the proportion of the three parental populations in each genome (light orange for South Asian, dark orange for Southeast Asian and blue for Northeast Asian). (DOCX) [file pntd.0006202.s001.docx]

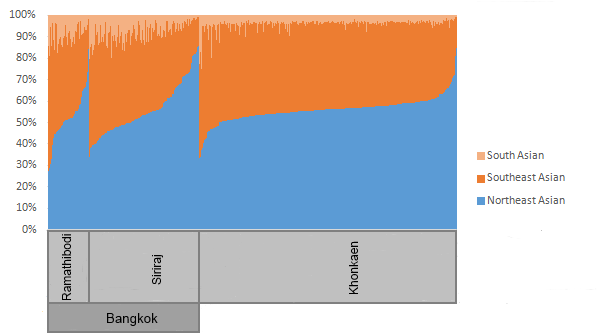


**S1 Figure.** **Global ancestry inferred through RFMix when using three parental ancestries (South, Northeast and Southeast Asian) for the three Thai hospital dengue cohorts.** Each vertical line represents an individual, and the three colours represent the proportion of the three parental populations in each genome (light orange for South Asian, dark orange for Southeast Asian and blue for Northeast Asian).
