# Supplementary material for: Joint ancestry and association test indicate two distinct pathogenic pathways involved in classical dengue fever and dengue shock syndrome
Source: PLoS Negl Trop Dis. 2018 Feb 15;12(2):e0006202. doi: 10.1371/journal.pntd.0006202 (PMC5813895; doi:10.1371/journal.pntd.0006202)
Supplement: S2 Fig — BMIX identified significant SNPs are indicated by a box. All SNPs have at least 5% minimum allele frequency in the population analysed. (DOCX) [file pntd.0006202.s002.docx]

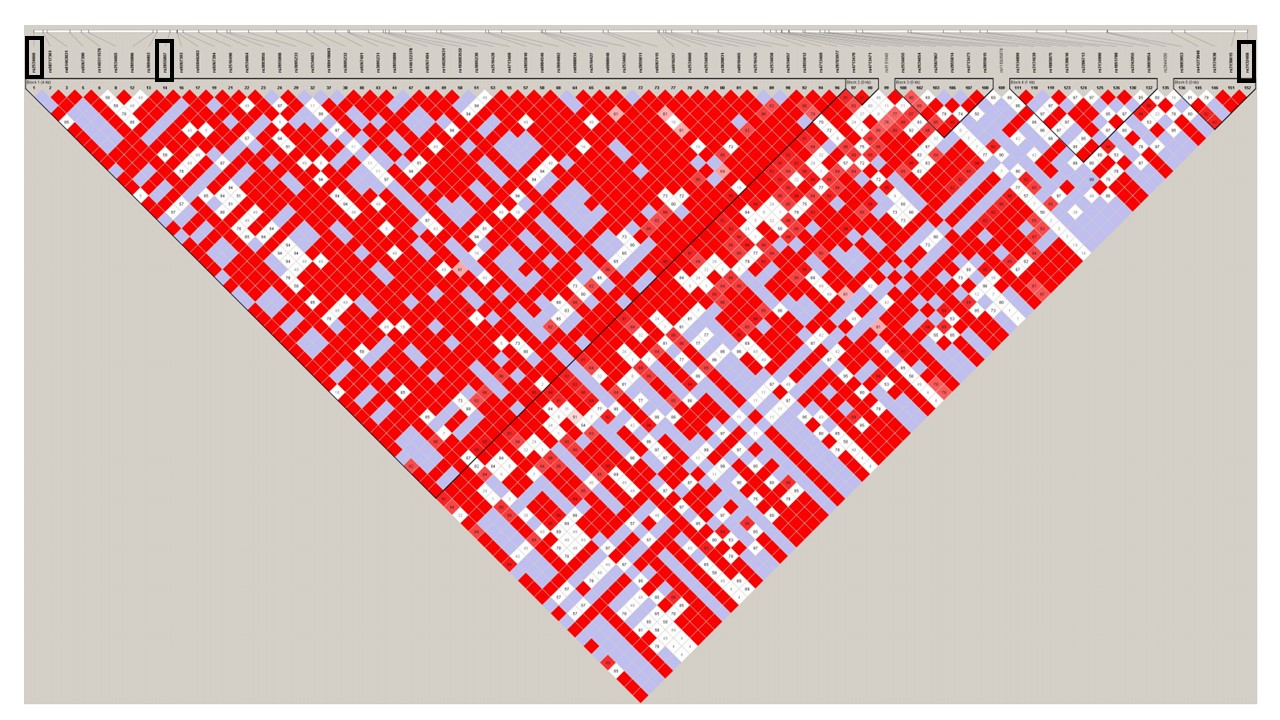


**S2 Figure.** **LD (D’) values for the *MICB* region in the Chinese population (CDX) from 1000 Genomes database.** BMIX identified significant SNPs are indicated by a box. All SNPs have at least 5% minimum allele frequency in the population analysed.
