# Supplementary material for: Joint ancestry and association test indicate two distinct pathogenic pathways involved in classical dengue fever and dengue shock syndrome
Source: PLoS Negl Trop Dis. 2018 Feb 15;12(2):e0006202. doi: 10.1371/journal.pntd.0006202 (PMC5813895; doi:10.1371/journal.pntd.0006202)
Supplement: S4 Fig — Locus zoom of the chromosomal region around genes (A–PLCB4, B–PLCE1, C–CHST10, D–AHRR, E–GRIP1, F–PPP2R5E) with significant p-values obtained for DSS and DF tests. The Asian recombination map was used. (DOCX) [file pntd.0006202.s004.docx]

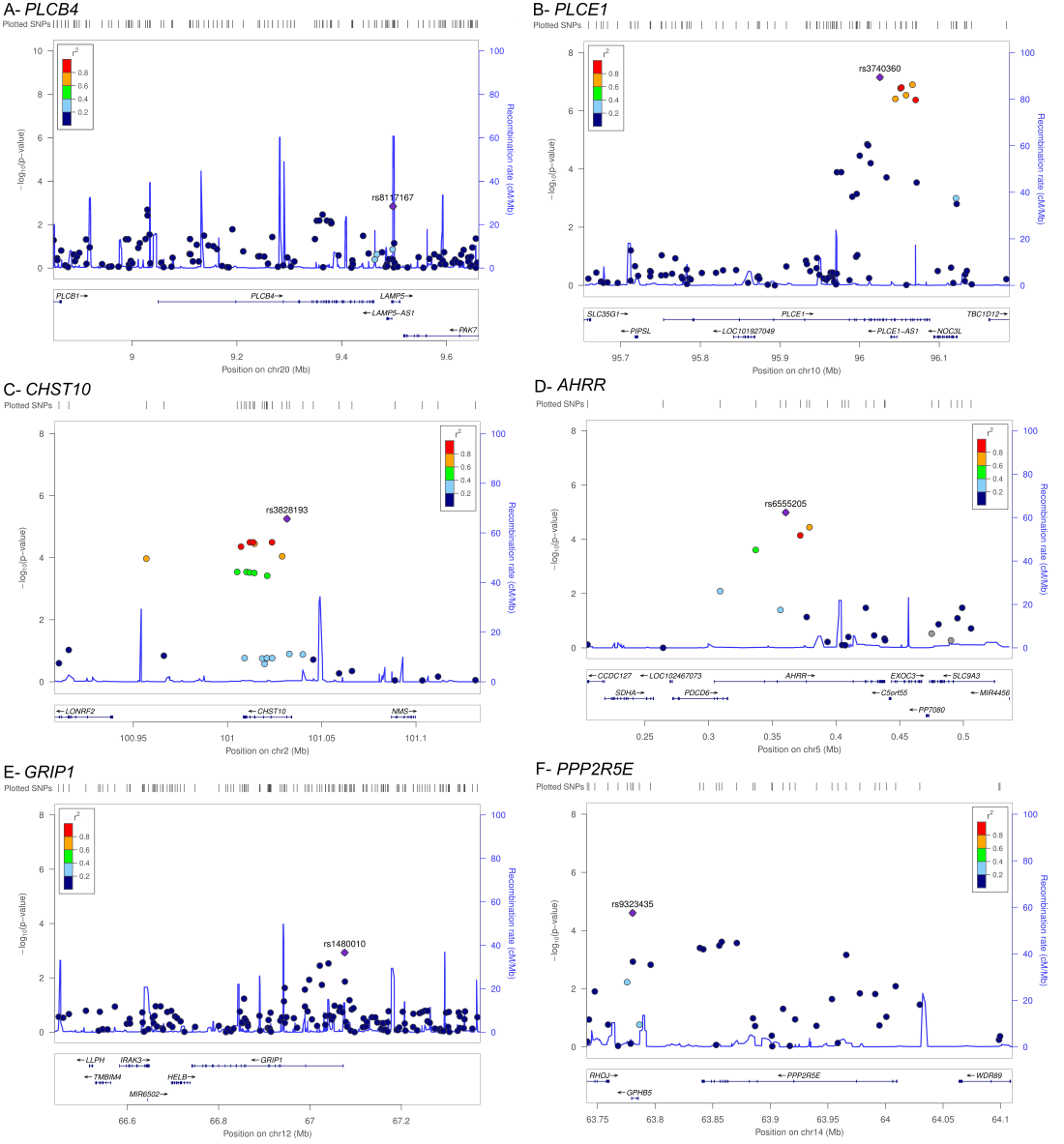


**S4 Figure.** **Locus zoom of the chromosomal region around genes (A – *PLCB4*, B – *PLCE1*, C – *CHST10*, D – *AHRR*, E – *GRIP1*, F – *PPP2R5E*) with significant p-values obtained for DSS and DF tests.** The Asian recombination map was used.
