# Supplementary material for: Joint ancestry and association test indicate two distinct pathogenic pathways involved in classical dengue fever and dengue shock syndrome
Source: PLoS Negl Trop Dis. 2018 Feb 15;12(2):e0006202. doi: 10.1371/journal.pntd.0006202 (PMC5813895; doi:10.1371/journal.pntd.0006202)
Supplement: S6 Fig — A–DSS test (p-values and D’ for SNPs surrounding the two spurious SNPs are highlighted). B—DF test. The red line indicates the significance threshold. (DOCX) [file pntd.0006202.s006.docx]

**
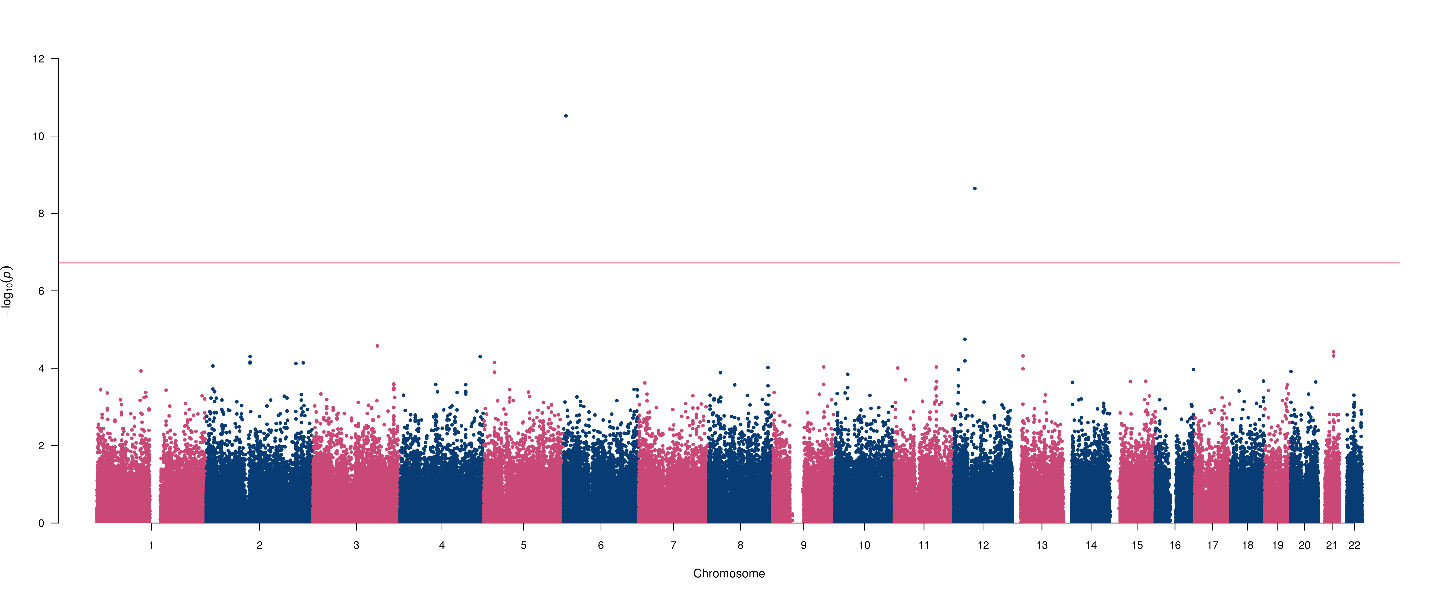
**

| Tag SNP | Chr | SNP | BP | Allele |  | OR | P-value | D' with tag SNP |
| --- | --- | --- | --- | --- | --- | --- | --- | --- |
|  | 6 | rs6939701 | 5934718 | C |  | 1.027 | 0.871 | 0.462 |
|  | 6 | rs3018 | 5938368 | T |  | 0.9635 | 0.8772 | 0.745 |
| **Tag** | **6** | **rs4959364** | **5947139** | **A** |  | **0.3191** | **3.00E-11** |  |
|  | 6 | rs11759185 | 5958615 | T |  | 0.977 | 0.8939 | 0.964 |
|  | 6 | rs1555536 | 5961601 | A |  | 0.9602 | 0.8128 | 0.830 |
|  | 12 | rs11168353 | 48408212 | T |  | 0.9491 | 0.8367 | 1.000 |
|  | 12 | rs12819124 | 48409054 | A |  | 0.68 | 0.1146 | 1.000 |
| **Tag** | **12** | **rs6580649** | **48410517** | **C** |  | **0.3528** | **2.26E-09** |  |
|  | 12 | rs11168357 | 48412138 | A |  | 1.167 | 0.3924 | 1.000 |
|  | 12 | rs7299271 | 48414058 | C |  | 1.074 | 0.6797 | 1.000 |

**A**

**
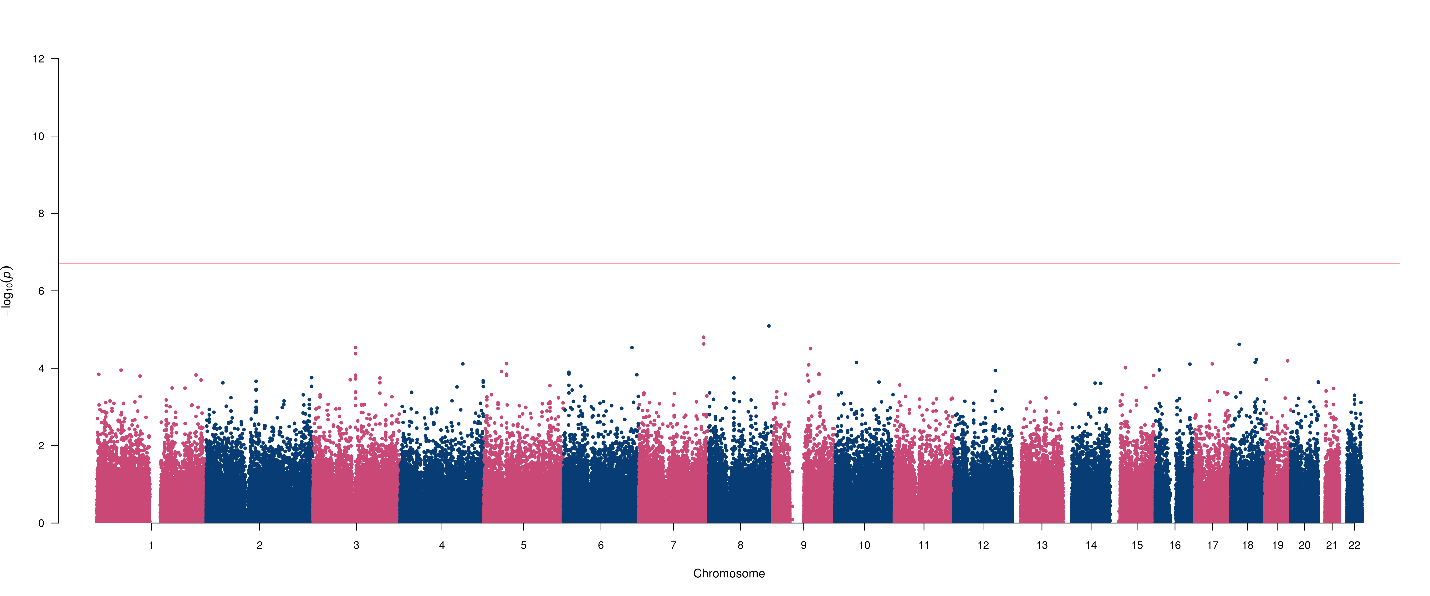
**

**B**

**S6 Figure Manhattan plots for the conventional association tests with PCA correction for population structure.** A – DSS test (p-values and D’ for SNPs surrounding the two spurious SNPs are highlighted). B - DF test. The red line indicates the significance threshold. (DOC)
