# Supplementary material for: Joint ancestry and association test indicate two distinct pathogenic pathways involved in classical dengue fever and dengue shock syndrome
Source: PLoS Negl Trop Dis. 2018 Feb 15;12(2):e0006202. doi: 10.1371/journal.pntd.0006202 (PMC5813895; doi:10.1371/journal.pntd.0006202)
Supplement: S10 Fig — Gene expression for CHST10 (A) and AHRR (B) in Thai dengue cohort along the course of disease from a transcriptome dataset for whole blood.[17] No significant differences in expression were observed. (DOCX) [file pntd.0006202.s010.docx]

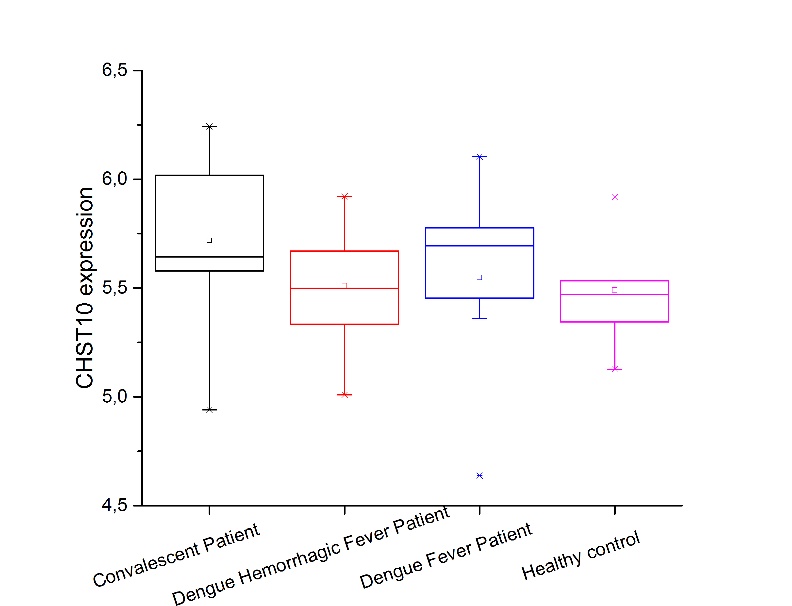


A


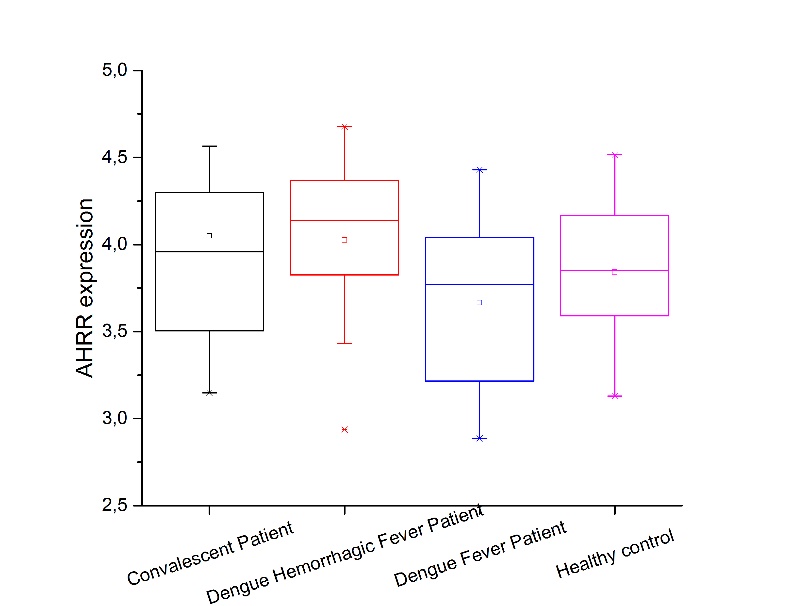


B

**S10 Figure.** **Gene expression for *CHST10* (A) and *AHRR* (B) in Thai dengue cohort along the course of disease from a transcriptome dataset for whole blood.**[**^1^**](#_ENREF_1) No significant differences in expression were observed.

**References**

1. Kwissa, M. *et al.* Dengue virus infection induces expansion of a CD14(+)CD16(+) monocyte population that stimulates plasmablast differentiation. *Cell Host Microbe* **16**, 115-27 (2014).
