# Supplementary material for: Joint ancestry and association test indicate two distinct pathogenic pathways involved in classical dengue fever and dengue shock syndrome
Source: PLoS Negl Trop Dis. 2018 Feb 15;12(2):e0006202. doi: 10.1371/journal.pntd.0006202 (PMC5813895; doi:10.1371/journal.pntd.0006202)
Supplement: S11 Fig — The protective alleles are indicated in green while the causative alleles are in red. (DOCX) [file pntd.0006202.s011.docx]

**
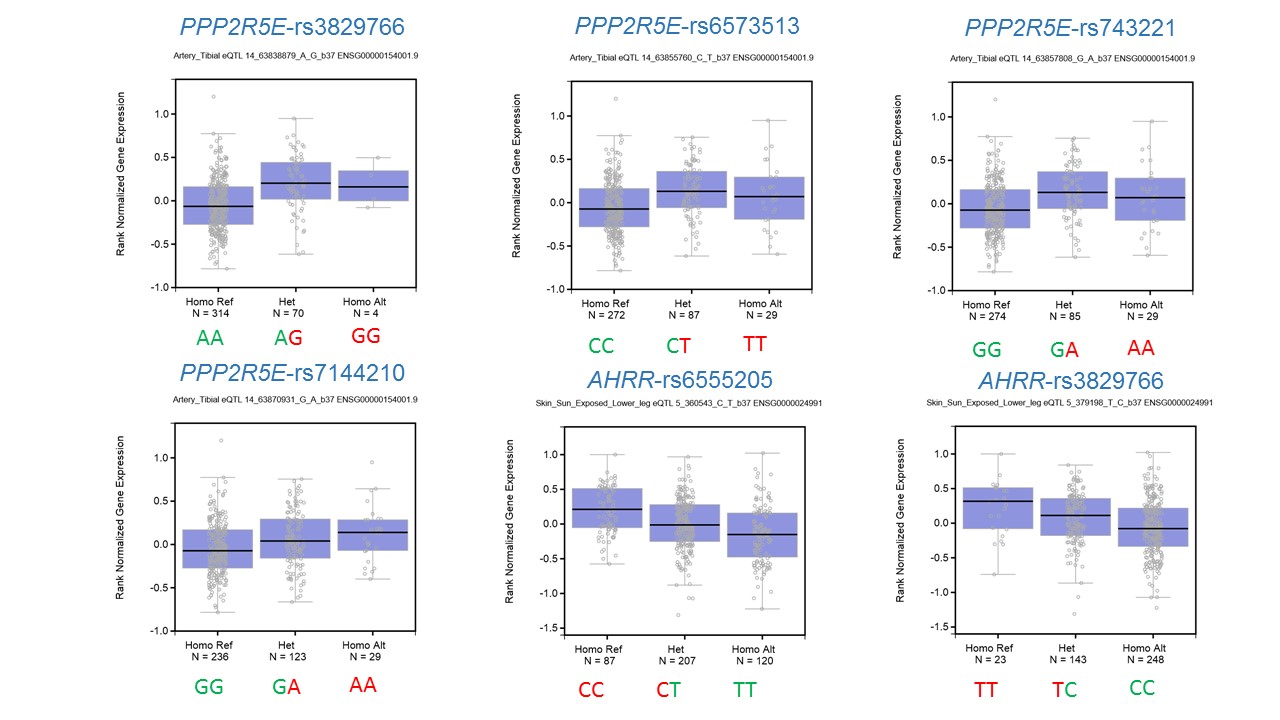
**

**S11 Figure mRNA expression profiles for the eQTLs in *PPP2R5E* and *AHRR* genes (information from GTEx database).** The protective alleles are indicated in green while the causative alleles are in red.
