# Supplementary figures and images for: Joint ancestry and association test indicate two distinct pathogenic pathways involved in classical dengue fever and dengue shock syndrome
Source: PLoS Negl Trop Dis. 2018 Feb 15;12(2):e0006202. doi: 10.1371/journal.pntd.0006202 (PMC5813895; doi:10.1371/journal.pntd.0006202)

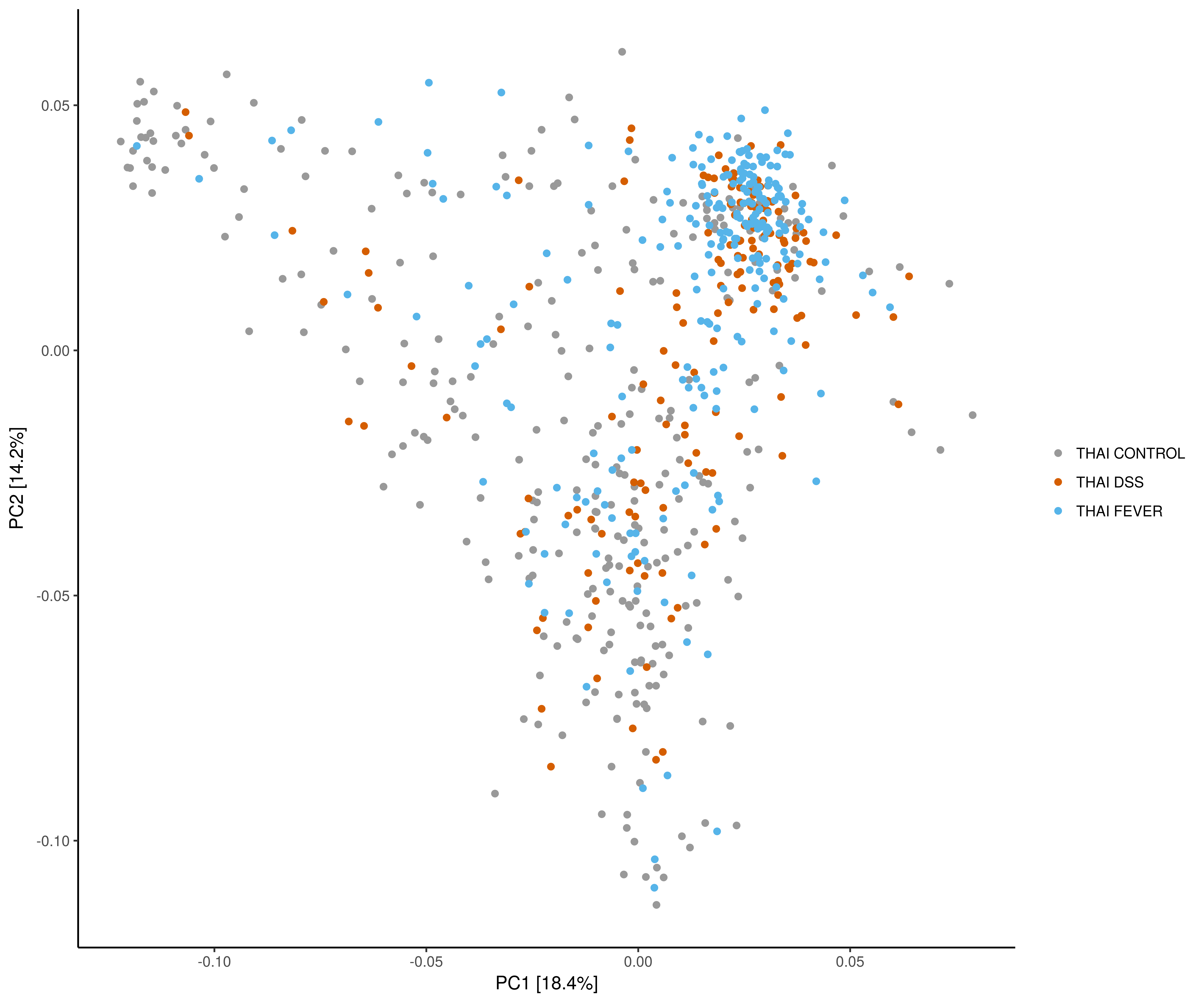


**S12 Fig.** **PCA of the Thai samples.** Plot of PC1 versus PC2 in the Thai control and patient cohorts.

Supplement: S12 Fig — Plot of PC1 versus PC2 in the Thai control and patient cohorts. (DOCX) [file pntd.0006202.s012.docx]
