# Supplementary material for: Joint ancestry and association test indicate two distinct pathogenic pathways involved in classical dengue fever and dengue shock syndrome
Source: PLoS Negl Trop Dis. 2018 Feb 15;12(2):e0006202. doi: 10.1371/journal.pntd.0006202 (PMC5813895; doi:10.1371/journal.pntd.0006202)
Supplement: S13 Fig — (DOCX) [file pntd.0006202.s013.docx]

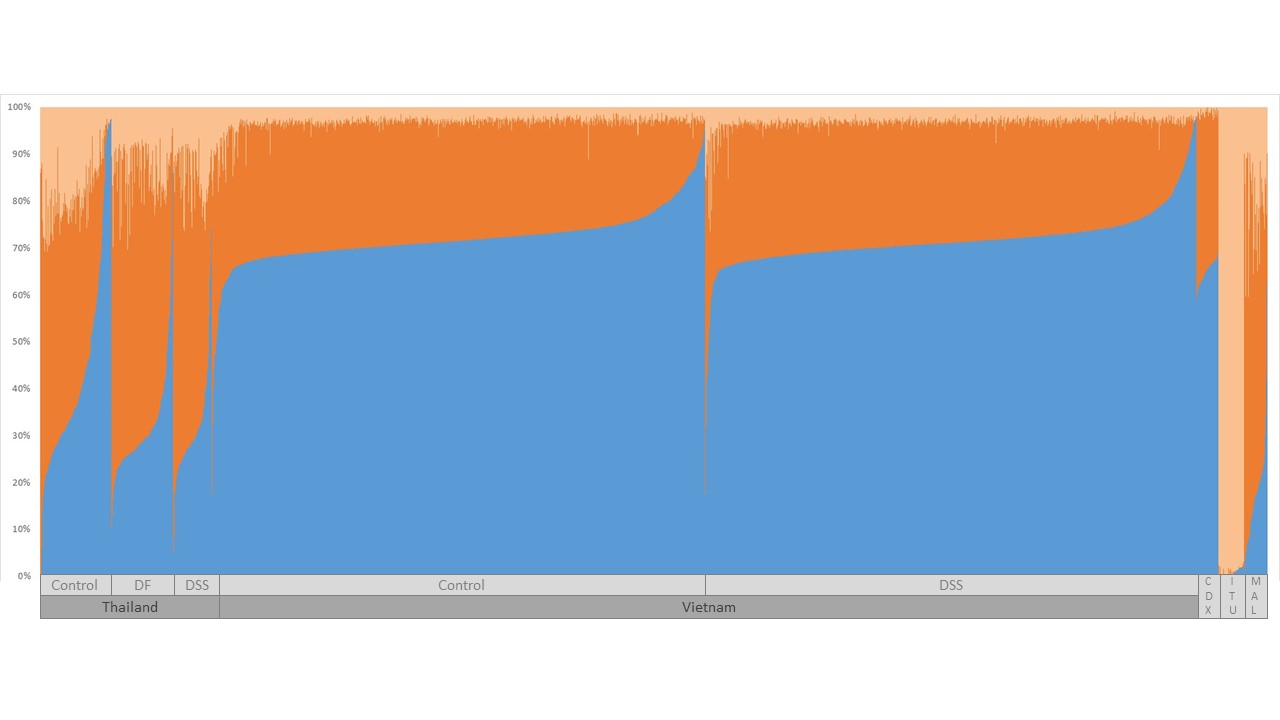


**S13 Fig. ADMIXTURE plot for K=3 for Thai and Vietnamese cohorts and the parental populations used in this work (CDX - Chinese Dai in Xishuangbanna; ITU- Indian Telugu from the UK (ITU); and MAL - Malaysian).**
