# Supplementary material for: Joint ancestry and association test indicate two distinct pathogenic pathways involved in classical dengue fever and dengue shock syndrome
Source: PLoS Negl Trop Dis. 2018 Feb 15;12(2):e0006202. doi: 10.1371/journal.pntd.0006202 (PMC5813895; doi:10.1371/journal.pntd.0006202)
Supplement: S1 Table — The base position refers to GRCh37 genome assembly. (DOCX) [file pntd.0006202.s014.docx]

**S1 Table.** **Significant SNPs in BMIX analysis for Vietnam DSS test.** The base position refers to GRCh37 genome assembly.

| Chr | SNP | BP | Allele | Association p-value | OR | BMIX posterior p-value in Northeast Asian ancestry | BMIX posterior p-value in Southeast Asian ancestry | Gene |
| --- | --- | --- | --- | --- | --- | --- | --- | --- |
| 4 | rs17603961 | 37386498 | A | 0.0007608 | 1.210 | 0.503 | 0.589 | *NWD2* |
| 6 | rs1051794 | 31379109 | G | 0.04197 | 1.195 | 0.896 | 0.762 | *MICA* |
| 6 | rs1131904 | 31383071 | G | 0.0002064 | 1.192 | 0.883 | 0.737 | *MICA/HCP5* |
| 6 | rs2534666 | 31468546 | A | 1.46E-08 | 1.343 | 0.993 | 0.999 | *MICB/Y_RNA* |
| 6 | rs2855807 | 31469323 | C | 9.38E-07 | 1.294 | 0.993 | 0.999 | *MICB/Y_RNA* |
| 6 | rs3132468 | 31475486 | C | 7.04E-08 | 1.398 | 0.999 | 1.000 | *MICB* |
| 6 | rs9267487 | 31511350 | G | 0.00121 | 1.442 |  | 0.637 | *DDX39B/DDX39B-AS1/ATP6V1G2/SNORD84/NFKBIL1* |
| 6 | rs3093662 | 31544189 | G | 0.001019 | 1.439 |  | 0.705 | *LTB/LTA/TNF* |
| 9 | rs2417485 | 106867106 | C | 0.001976 | 1.193 | 0.821 | 0.837 | *SMC2* |
| 9 | rs2122576 | 106870187 | C | 0.002373 | 1.191 | 0.854 | 0.842 | *SMC2* |
| 10 | rs3740360 | 96025491 | C | 7.05E-08 | 0.756 | 0.529 | 0.649 | *PLCE1* |
| 10 | rs2274223 | 96066341 | G | 1.26E-07 | 0.768 |  | 0.504 | *PLCE1* |
| 10 | rs2421027 | 124203648 | G | 5.16E-05 | 0.496 |  | 0.550 |  |
| 12 | rs12317948 | 31884832 | A | 0.001309 | 1.224 | 0.523 |  | *AMN1* |
| 20 | rs6074355 | 11786106 | A | 0.0001216 | 1.506 | 0.776 | 0.887 | *LINC00687/AL080274.1* |
| 20 | rs6074356 | 11786432 | A | 0.0001268 | 1.512 | 0.773 | 0.885 | *LINC00687/AL080274.1* |
