# Supplementary material for: Joint ancestry and association test indicate two distinct pathogenic pathways involved in classical dengue fever and dengue shock syndrome
Source: PLoS Negl Trop Dis. 2018 Feb 15;12(2):e0006202. doi: 10.1371/journal.pntd.0006202 (PMC5813895; doi:10.1371/journal.pntd.0006202)
Supplement: S2 Table — The base position refers to GRCh37 genome assembly. (DOCX) [file pntd.0006202.s015.docx]

**S2 Table.** **Significant SNPs in BMIX analysis for Thai DSS vs control test.** The base position refers to GRCh37 genome assembly.

| Chr | SNP | BP | Allele | Association p-value | OR | BMIX posterior p-value in Northeast Asian ancestry | BMIX posterior p-value in Southeast Asian ancestry | Gene |
| --- | --- | --- | --- | --- | --- | --- | --- | --- |
| 1 | rs705731 | 202298894 | C | 2.09E-05 | 0.497 | 0.585 |  | *UBE2T* |
| 2 | rs1884725 | 31571786 | A | 0.001065 | 1.778 | 0.628 |  | *XDH* |
| 2 | rs7566302 | 33595197 | A | 0.002199 | 0.576 |  | 0.583 | *LTBP1* |
| 2 | rs11679130 | 35730792 | C | 0.0008383 | 1.605 |  | 0.569 |  |
| 2 | rs11682759 | 204192201 | C | 5.15E-05 | 1.767 | 0.541 |  | *ABI2/RP11-363J17.1* |
| 2 | rs13383306 | 230179536 | A | 0.0006001 | 2.159 | 0.522 |  |  |
| 4 | rs17256627 | 11873033 | T | 9.81E-05 | 0.401 | 0.924 | 0.852 |  |
| 4 | rs11937407 | 81413618 | C | 3.00E-05 | 0.538 | 0.866 |  | *C4orf22* |
| 4 | rs13109014 | 85260011 | T | 2.01E-06 | 2.446 | 0.961 |  |  |
| 5 | rs1501938 | 24059579 | G | 4.08E-05 | 2.004 | 0.833 | 0.643 | *C5orf17* |
| 5 | rs6452189 | 24163109 | T | 1.41E-05 | 1.974 | 0.936 | 0.655 | *C5orf17* |
| 6 | rs4959364 | 5947139 | A | 7.22E-17 | 0.285 | 1.000 | 1.000 |  |
| 8 | rs10105057 | 19614339 | C | 0.0007059 | 1.622 |  | 0.679 | *CSGALNACT1* |
| 8 | rs7837390 | 19626120 | C | 0.00057 | 1.632 |  | 0.734 |  |
| 8 | rs6983707 | 91133678 | G | 0.0001822 | 1.706 | 0.688 |  |  |
| 9 | rs16922639 | 106004562 | T | 2.56E-05 | 2.476 |  | 0.958 | *RP11-341A22.2* |
| 12 | rs6580649 | 48410517 | C | 2.26E-11 | 0.357 | 1.000 | 1.000 | *RP1-228P16.4* |
| 20 | rs16995800 | 9352562 | G | 0.006618 | 0.565 | 0.703 |  | *PLCB4* |
| 20 | rs2299676 | 9357437 | A | 0.006497 | 0.582 | 0.542 |  | *PLCB4* |
| 20 | rs7269910 | 9363565 | G | 0.003453 | 0.553 | 0.917 |  | *PLCB4* |
| 20 | rs1997696 | 9378671 | A | 0.007033 | 0.667 | 0.691 |  | *PLCB4* |
| 20 | rs6133707 | 9379949 | G | 0.008858 | 0.675 | 0.679 |  | *PLCB4* |
| 20 | rs6056595 | 9380556 | G | 0.008289 | 0.672 | 0.691 |  | *PLCB4* |
