# Supplementary material for: Joint ancestry and association test indicate two distinct pathogenic pathways involved in classical dengue fever and dengue shock syndrome
Source: PLoS Negl Trop Dis. 2018 Feb 15;12(2):e0006202. doi: 10.1371/journal.pntd.0006202 (PMC5813895; doi:10.1371/journal.pntd.0006202)
Supplement: S3 Table — The base position refers to GRCh37 genome assembly. (DOCX) [file pntd.0006202.s016.docx]

**S3 Table. Significant SNPs in BMIX analysis for Thai DF test.** The base position refers to GRCh37 genome assembly.

| Chr | SNP | BP | Allele | Association p-value | OR | BMIX posterior p-value in Northeast Asian ancestry | BMIX posterior p-value in Southeast Asian ancestry | Gene |
| --- | --- | --- | --- | --- | --- | --- | --- | --- |
| 1 | rs12028426 | 171927696 | T | 0.0003996 | 1.579 |  | 0.938 | *DNM3* |
| 2 | rs394874 | 17655480 | T | 3.17E-05 | 0.387 |  | 0.891 |  |
| 2 | rs2309798 | 100956949 | G | 0.0001066 | 0.594 |  | 0.826 |  |
| 2 | rs4850931 | 101005145 | C | 0.0002867 | 0.583 |  | 0.556 | *CHST10* |
| 2 | rs1030902 | 101007178 | T | 4.40E-05 | 0.577 |  | 0.940 | *CHST10* |
| 2 | rs2241811 | 101011724 | C | 0.0003 | 0.582 |  | 0.565 | *CHST10* |
| 2 | rs2241810 | 101011877 | T | 3.19E-05 | 0.571 |  | 0.957 | *CHST10* |
| 2 | rs4149518 | 101013649 | A | 3.19E-05 | 0.571 |  | 0.957 | *CHST10* |
| 2 | rs2241809 | 101014363 | C | 3.54E-05 | 0.573 |  | 0.854 | *CHST10* |
| 2 | rs4149510 | 101023635 | G | 3.19E-05 | 0.571 |  | 0.957 | *CHST10* |
| 2 | rs4851313 | 101029002 | G | 8.99E-05 | 0.561 |  | 0.835 | *CHST10* |
| 2 | rs3828193 | 101031561 | G | 5.57E-06 | 0.545 | 0.794 | 0.987 | *CHST10* |
| 5 | rs6555205 | 360543 | T | 1.03E-05 | 0.510 |  | 0.851 | *AHRR* |
| 5 | rs2721020 | 379198 | C | 3.61E-05 | 0.558 |  | 0.505 | *AHRR* |
| 5 | rs1994929 | 23507631 | T | 0.0008452 | 1.666 | 0.780 |  | *PRDM9* |
| 5 | rs7708103 | 177410416 | G | 0.001826 | 0.664 |  | 0.648 | *RP11-1252I4.2* |
| 6 | rs532098 | 32578052 | A | 8.60E-05 | 0.612 | 0.597 |  |  |
| 6 | rs9397270 | 156163798 | T | 6.82E-08 | 2.019 | 0.864 |  |  |
| 8 | rs2255522 | 137523980 | G | 5.23E-06 | 0.566 | 0.910 | 0.976 | *RP11-431D12.1* |
| 12 | rs1480010 | 67076016 | T | 0.001176 | 0.557 | 0.929 |  | *GRIP1* |
| 12 | rs2717418 | 70973596 | G | 0.001935 | 1.524 | 0.845 |  | *PTPRB* |
| 14 | rs9323435 | 63780460 | C | 2.49E-05 | 1.696 |  | 0.853 | *GPHB5* |
| 14 | rs3829766 | 63838879 | G | 0.0003975 | 1.547 |  | 0.527 | *PPP2R5E* |
| 14 | rs6573513 | 63855760 | C | 0.0003278 | 0.638 |  | 0.662 | *PPP2R5E* |
| 14 | rs743221 | 63857808 | G | 0.0002449 | 0.632 |  | 0.718 | *PPP2R5E* |
| 14 | rs7144210 | 63870931 | G | 0.000268 | 0.633 |  | 0.843 | *PPP2R5E* |
| 16 | rs7184164 | 64281609 | C | 0.002642 | 1.847 |  | 0.576 | *AC012322.1* |
| 21 | rs2212870 | 20941913 | T | 0.01392 | 1.355 | 0.545 |  |  |
| 21 | rs2825968 | 21415693 | C | 0.004167 | 1.421 | 0.887 |  |  |
| 21 | rs2825993 | 21443895 | G | 0.001717 | 0.622 | 0.573 |  |  |
| 21 | rs2826059 | 21543190 | T | 0.0003941 | 0.459 | 0.738 | 0.549 |  |
