# Supplementary material for: Joint ancestry and association test indicate two distinct pathogenic pathways involved in classical dengue fever and dengue shock syndrome
Source: PLoS Negl Trop Dis. 2018 Feb 15;12(2):e0006202. doi: 10.1371/journal.pntd.0006202 (PMC5813895; doi:10.1371/journal.pntd.0006202)
Supplement: S4 Table — (DOCX) [file pntd.0006202.s017.docx]

**S4 Table.** **Annotation of the significant SNPs in BMIX analysis for Vietnam DSS test, inferred by using the Variant Effect Predictor (VEP) tool from Ensemble.**

| Chr | SNP | Impact in VEP | Gene | Consequence |
| --- | --- | --- | --- | --- |
| 4 | rs17603961 | Modifier | *NWD2* | intron variant |
| 6 | rs1051794 | Moderate/Modifier | *MICA* | missense, intron, non-coding transcript variant |
| 6 | rs1131904 | Modifier | *MICA/HCP5* | 3 prime UTR, intron, non-coding transcript variant |
| 6 | rs2534666 | Modifier | *MICB/Y_RNA* | downstream gene, intron, upstream gene, regulatory region (enhancer) variant |
| 6 | rs2855807 | Modifier | *MICB/Y_RNA* | downstream gene, intron, upstream gene variant |
| 6 | rs3132468 | Modifier | *MICB* | downstream gene, intron variant |
| 6 | rs9267487 | Modifier | *DDX39B/DDX39B-AS1/ATP6V1G2/SNORD84/NFKBIL1* | upstream gene, downstream gene, intron, NMD transcript, regulatory region (promoter) variant |
| 6 | rs3093662 | Modifier | *LTB/LTA/TNF* | downstream gene, intron, regulatory region (promoter) variant |
| 9 | rs2417485 | Modifier | *SMC2* | intron, downstream gene variant |
| 9 | rs2122576 | Modifier | *SMC2* | intron variant |
| 10 | rs3740360 | Modifier | *PLCE1* | intron variant |
| 10 | rs2274223 | Moderate | *PLCE1* | missense variant |
| 10 | rs2421027 | Modifier |  | intergenic variant |
| 12 | rs12317948 | Modifier | *AMN1* | upstream gene variant |
| 20 | rs6074355 | Modifier | *LINC00687/AL080274.1* | downstream gene, intron, non-coding transcript variant |
| 20 | rs6074356 | Modifier | *LINC00687/AL080274.1* | downstream gene, intron, non-coding transcript variant |
