# Supplementary material for: Joint ancestry and association test indicate two distinct pathogenic pathways involved in classical dengue fever and dengue shock syndrome
Source: PLoS Negl Trop Dis. 2018 Feb 15;12(2):e0006202. doi: 10.1371/journal.pntd.0006202 (PMC5813895; doi:10.1371/journal.pntd.0006202)
Supplement: S5 Table — (DOCX) [file pntd.0006202.s018.docx]

**S5 Table.** **Annotation of the significant SNPs in BMIX analysis for Thai DSS test, inferred by using the Variant Effect Predictor (VEP) tool from Ensemble.**

| Chr | SNP | Impact in VEP | Gene | Consequence |
| --- | --- | --- | --- | --- |
| 1 | rs705731 | Modifier | *UBE2T* | downstream gene variant |
| 2 | rs1884725 | Low | *XDH* | synonymous variant |
| 2 | rs7566302 | Modifier | *LTBP1* | intron variant |
| 2 | rs11679130 | Modifier |  | intergenic variant |
| 2 | rs11682759 | Modifier | *ABI2/RP11-363J17.1* | upstream gene, downstream gene, regulatory region (promoter) variant |
| 2 | rs13383306 | Modifier |  | intergenic variant |
| 4 | rs17256627 | Modifier |  | intergenic variant |
| 4 | rs11937407 | Modifier | *C4orf22* | intron, non-coding transcript, NMD transcript variant |
| 4 | rs13109014 | Modifier |  | intergenic variant |
| 5 | rs1501938 | Modifier | *C5orf17* | intron variant, NMD transcript variant |
| 5 | rs6452189 | Modifier | *C5orf17* | intron, NMD transcript, regulatory region (promoter flanking region) variant |
| 6 | rs4959364 | Modifier |  | intergenic variant |
| 8 | rs10105057 | Modifier | *CSGALNACT1* | intron, non-coding transcript, regulatory region (promoter) variant |
| 8 | rs7837390 | Modifier |  | intergenic variant |
| 8 | rs6983707 | Modifier |  | intergenic variant |
| 9 | rs16922639 | Modifier | *RP11-341A22.2* | intron, non-coding transcript variant |
| 12 | rs6580649 | Modifier | *RP1-228P16.4* | intron, non-coding, downstream gene variant |
| 20 | rs16995800 | Modifier | *PLCB4* | intron, non-coding transcript variant |
| 20 | rs2299676 | Modifier | *PLCB4* | intron, non-coding transcript variant |
| 20 | rs7269910 | Modifier | *PLCB4* | intron, non-coding transcript variant |
| 20 | rs1997696 | Modifier | *PLCB4* | intron, non-coding transcript, regulatory region (promoter flanking region) variant |
| 20 | rs6133707 | Modifier | *PLCB4* | intron, non-coding transcript, regulatory region (promoter flanking region) variant |
| 20 | rs6056595 | Modifier | *PLCB4* | intron, non-coding transcript, regulatory region (promoter flanking region) variant |
