# Supplementary material for: Joint ancestry and association test indicate two distinct pathogenic pathways involved in classical dengue fever and dengue shock syndrome
Source: PLoS Negl Trop Dis. 2018 Feb 15;12(2):e0006202. doi: 10.1371/journal.pntd.0006202 (PMC5813895; doi:10.1371/journal.pntd.0006202)
Supplement: S6 Table — (DOCX) [file pntd.0006202.s019.docx]

**S6 Table.** **Annotation of the significant SNPs in BMIX analysis for Thai DF test, inferred by using the Variant Effect Predictor (VEP) tool from Ensemble.**

| Chr | SNP | Impact in VEP | Gene | Consequence |
| --- | --- | --- | --- | --- |
| 1 | rs12028426 | Modifier | DNM3 | intron variant |
| 2 | rs394874 | Modifier |  | intergenic variant |
| 2 | rs2309798 | Modifier |  | intergenic variant |
| 2 | rs4850931 | Modifier | CHST10 | downstream gene variant |
| 2 | rs1030902 | Modifier | CHST10 | downstream gene variant |
| 2 | rs2241811 | Modifier | CHST10 | downstream gene, intron, regulatory region (TF binding site) variant |
| 2 | rs2241810 | Modifier | CHST10 | downstream gene, intron, regulatory region (TF binding site) variant |
| 2 | rs4149518 | Modifier | CHST10 | downstream gene, intron variant |
| 2 | rs2241809 | Modifier | CHST10 | downstream gene, splice region, intron variant |
| 2 | rs4149510 | Modifier | CHST10 | intron, downstream gene, non-coding transcript variant |
| 2 | rs4851313 | Modifier | CHST10 | intron, downstream gene, non-coding transcript variant |
| 2 | rs3828193 | Modifier | CHST10 | 5 prime UTR, non-coding transcript exon, non-coding transcript variant |
| 5 | rs6555205 | Modifier | AHRR | intron, regulatory region (open chromatin region) variant |
| 5 | rs2721020 | Modifier | AHRR | intron, regulatory region (promoter flanking region) variant |
| 5 | rs1994929 | Modifier | PRDM9 | intron, upstream gene variant |
| 5 | rs7708103 | Modifier | RP11-1252I4.2 | downstream gene variant |
| 6 | rs532098 | Modifier |  | intergenic variant |
| 6 | rs9397270 | Modifier |  | intergenic variant |
| 8 | rs2255522 | Modifier | RP11-431D12.1 | intron, non-coding transcript variant |
| 12 | rs1480010 | Modifier | GRIP1 | intron, upstream gene variant |
| 12 | rs2717418 | Modifier | PTPRB | intron variant |
| 14 | rs9323435 | Modifier | GPHB5 | intron, non-coding transcript variant |
| 14 | rs3829766 | Modifier | PPP2R5E | downstream gene, 3 prime UTR variant |
| 14 | rs6573513 | Modifier | PPP2R5E | intron, non-coding transcript, regulatory region (CTCF and TF binding sites) variant |
| 14 | rs743221 | Modifier | PPP2R5E | intron, non-coding transcript variant |
| 14 | rs7144210 | Modifier | PPP2R5E | intron, non-coding transcript variant |
| 16 | rs7184164 | Modifier | AC012322.1 | intron, non-coding transcript variant |
| 21 | rs2212870 | Modifier |  | intergenic variant |
| 21 | rs2825968 | Modifier |  | intergenic variant |
| 21 | rs2825993 | Modifier |  | intergenic variant |
| 21 | rs2826059 | Modifier |  | intergenic variant |
