# Supplementary material for: Joint ancestry and association test indicate two distinct pathogenic pathways involved in classical dengue fever and dengue shock syndrome
Source: PLoS Negl Trop Dis. 2018 Feb 15;12(2):e0006202. doi: 10.1371/journal.pntd.0006202 (PMC5813895; doi:10.1371/journal.pntd.0006202)
Supplement: S7 Table — (DOCX) [file pntd.0006202.s020.docx]

**S7 Table.** **Association p-values in the entire Thai cohort in six and two SNPs selected from the sets of BMIX-associated SNPs with DF and DSS phenotypes, respectively.**

|  |  |  |  | GWAS Discovery | | | | Total cohort | | | |
| --- | --- | --- | --- | --- | --- | --- | --- | --- | --- | --- | --- |
| Phenotype | SNP (alleles) | Chromosome (position) HG37 | Candidate gene | MAF cases | MAF controls | OR | P-value | MAF cases | MAF controls | OR | P-value |
| DF | rs2241811 (G/A) | 2 (101011724) | CHST10 | 0.175 | 0.268 | 0.582 | 0.0003 | 0.181 | 0.256 | 0.643 | 0.0004132 |
| DF | rs2241809 (G/A) | 2 (101014363) | CHST10 | 0.248 | 0.365 | 0.573 | 3.54E-05 | 0.248 | 0.343 | 0.629 | 3.84E-05 |
| DF | rs6555205 (A/G) | 5 (360543) | AHRR | 0.157 | 0.265 | 0.510 | 1.03E-05 | 0.157 | 0.249 | 0.562 | 1.02E-05 |
| DF | rs1480010 (A/G) | 12 (67076016) | GRIP1 | 0.105 | 0.174 | 0.557 | 0.001176 | 0.098 | 0.176 | 0.508 | 1.35E-05 |
| DF | rs6573513 (G/A) | 14 (63855760) | PPP2R5E | 0.349 | 0.459 | 0.638 | 0.0003278 | 0.359 | 0.447 | 0.691 | 0.0004071 |
| DF | rs7144210 (G/A) | 14 (63870931) | PPP2R5E | 0.347 | 0.457 | 0.633 | 0.000268 | 0.354 | 0.453 | 0.661 | 8.21E-05 |
| DSS | rs7269910 (G/A) | 20 (9363565) | PLCB4 | 0.116 | 0.195 | 0.553 | 0.003453 | 0.123 | 0.193 | 0.585 | 0.002431 |
| DSS | rs1997696 (A/C) | 20 (9378671) | PLCB4 | 0.289 | 0.381 | 0.667 | 0.007033 | 0.327 | 0.493 | 0.500 | 4.74E-08 |
