# Supplementary material for: Joint ancestry and association test indicate two distinct pathogenic pathways involved in classical dengue fever and dengue shock syndrome
Source: PLoS Negl Trop Dis. 2018 Feb 15;12(2):e0006202. doi: 10.1371/journal.pntd.0006202 (PMC5813895; doi:10.1371/journal.pntd.0006202)
Supplement: S8 Table — (DOCX) [file pntd.0006202.s021.docx]

**S8 Table. Detailed identification of motif sequences across DENV serotypes.**

| DENV serotype | Motif type | Motif sequence | Start | Stop | DENV protein |
| --- | --- | --- | --- | --- | --- |
| DENV1 | LxxLxE | LGELCEDTM | 155 | 163 | ER anchor for the protein C |
| DENV1 | MxxIxE | MFLIAENKI | 1333 | 1341 |  |
| DENV1 | LxxLxE | LTTLWEGSP | 2446 | 2454 | NS4B |
| DENV1 | LxxVxE | LKKVTEVKG | 2587 | 2595 | NS5 |
| DENV1 | LxxIxE | LCDIGESSP | 2637 | 2645 | NS5 |
| DENV1 | IxxIxE | ITDIMEPEH | 3045 | 3053 | NS5 |
| DENV2 | LxxLxE | LGELCEDTI | 155 | 163 | ER anchor for the protein C |
| DENV2 | LxxIxE | LNLITEMGR | 2097 | 2105 | NS4A |
| DENV2 | LxxLxE | LSELPETLE | 2138 | 2146 | NS4A |
| DENV2 | LxxVxE | LKNVREVKG | 2585 | 2593 | NS5 |
| DENV2 | LxxIxE | LCDIGESSP | 2635 | 2643 | NS5 |
| DENV2 | LxxLxE | LGFLNEDHW | 2980 | 2988 | NS5 |
| DENV3 | CxxIxE | CPHITEVEP | 167 | 175 | ER anchor for the protein C |
| DENV3 | IxxIxE | IRPINEKEE | 1108 | 1116 | NS1 |
| DENV3 | LxxLxE | LAILFEEVM | 1140 | 1148 | NS2A |
| DENV3 | LxxVxE | LDLVTEIGR | 2096 | 2104 | NS4A |
| DENV3 | LxxVxE | LKKVTEVRG | 2584 | 2592 | NS5 |
| DENV3 | LxxIxE | LCDIGESSP | 2634 | 2642 | NS5 |
| DENV3 | MxxIxE | MDVIGERIK | 2763 | 2771 | NS5 |
| DENV3 | IxxIxE | IKRIKEEHN | 2770 | 2778 | NS5 |
| DENV3 | LxxLxE | LGFLNEDHW | 2978 | 2986 | NS5 |
| DENV4 | LxxLxE | LRFLGEDGC | 1095 | 1103 | NS1 |
| DENV4 | IxxIxE | IRDVEETNM | 1430 | 1438 | NS2B |
| DENV4 | LxxLxE | LDILTEIAS | 2096 | 2104 | NS4A |
| DENV4 | LxxLxE | LNELPESLE | 2137 | 2145 | NS4A |
| DENV4 | LxxVxE | LLWVAEIQP | 2186 | 2194 | NS4A |
| DENV4 | IxxIxE | IRWIVERGM | 2549 | 2557 | NS5 |
| DENV4 | LxxVxE | LKNVTEVKG | 2581 | 2589 | NS5 |
| DENV4 | LxxIxE | LCDIGESSS | 2631 | 2639 | NS5 |
| DENV4 | LxxLxE | LQRLQEEHK | 2768 | 2776 | NS5 |
| DENV4 | LxxLxE | LGFLNEDHW | 2976 | 2984 | NS5 |
| DENV4 | LxxIxE | LGYILEEID | 3002 | 3010 | NS5 |
