# Supplementary material for: Joint ancestry and association test indicate two distinct pathogenic pathways involved in classical dengue fever and dengue shock syndrome
Source: PLoS Negl Trop Dis. 2018 Feb 15;12(2):e0006202. doi: 10.1371/journal.pntd.0006202 (PMC5813895; doi:10.1371/journal.pntd.0006202)
Supplement: S9 Table — (DOCX) [file pntd.0006202.s022.docx]

**S9 Table. Information on DENV serotype and primary/secondary infection in the Thai cohort.**

|  | **DF** | **DSS** |
| --- | --- | --- |
| **Serotype** |  |  |
| DENV1 | 85 | 21 |
| DENV2 | 38 | 36 |
| DENV3 | 15 | 3 |
| DENV4 | 41 | 9 |
| Not available | 73 | 90 |
| **Immune Status** |  |  |
| Primary | 32 | 6 |
| Secondary | 194 | 135 |
| Not available | 26 | 18 |
