# Supplementary material for: Joint ancestry and association test indicate two distinct pathogenic pathways involved in classical dengue fever and dengue shock syndrome
Source: PLoS Negl Trop Dis. 2018 Feb 15;12(2):e0006202. doi: 10.1371/journal.pntd.0006202 (PMC5813895; doi:10.1371/journal.pntd.0006202)
Supplement: S10 Table — (DOCX) [file pntd.0006202.s023.docx]

**S10 Table. Primers used for mutagenesis and DNA assembly protocols.**

| **Gene** | **Description** | **Forward Primer (5’-3')** | **Reverse Primer (5’-3')** |
| --- | --- | --- | --- |
| DENV2-NS5 | Mutagenesis | AATCCCACGGTAGAAGCA | TGTGTCACACTTTTCTGG |
| DENV1-NS5 | Assembly - insert | GCTTGGTACCATGGGCACGGGAGCCCAA | CTCCACCCCCCCAGAGTGCCCCTTCGGG |
| DENV1-NS5 | Assembly - plasmid | GGCACTCTGGGGGGGTGGAGGCTCTGATAG | CCGTGCCCATGGTACCAAGCTTGGTGGCGG |
