# Supplementary material for: Joint ancestry and association test indicate two distinct pathogenic pathways involved in classical dengue fever and dengue shock syndrome
Source: PLoS Negl Trop Dis. 2018 Feb 15;12(2):e0006202. doi: 10.1371/journal.pntd.0006202 (PMC5813895; doi:10.1371/journal.pntd.0006202)

| Chr | SNP | BP | Allele | Association p-value | OR | BMIX posterior p-value in Northeast Asian ancestry | BMIX posterior p-value in Southeast Asian ancestry | Gene |
| --- | --- | --- | --- | --- | --- | --- | --- | --- |
| **RUN 1** | | | | | | | | |
| 7 | rs7799285 | 100968363 | G | 0.0009861 | 0.6005 |  | 0.5479738 | *RABL5* |
| 9 | rs12683380 | 9929916 | A | 0.0005549 | 1.629 | 0.8485055 |  | *PTPRD* |
| 21 | rs2828104 | 24764752 | C | 7.276e-05 | 1.746 | 0.5206194 | 0.7748023 | *EEF1A1P1* |

Association


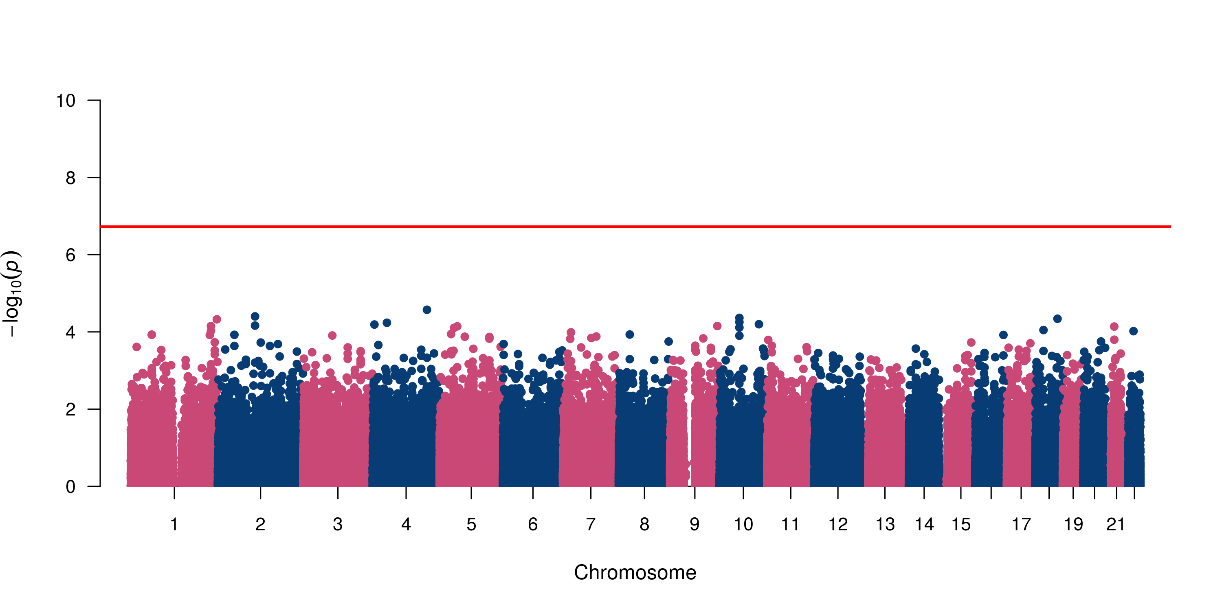


BMIX – NORTHEAST ASIAN


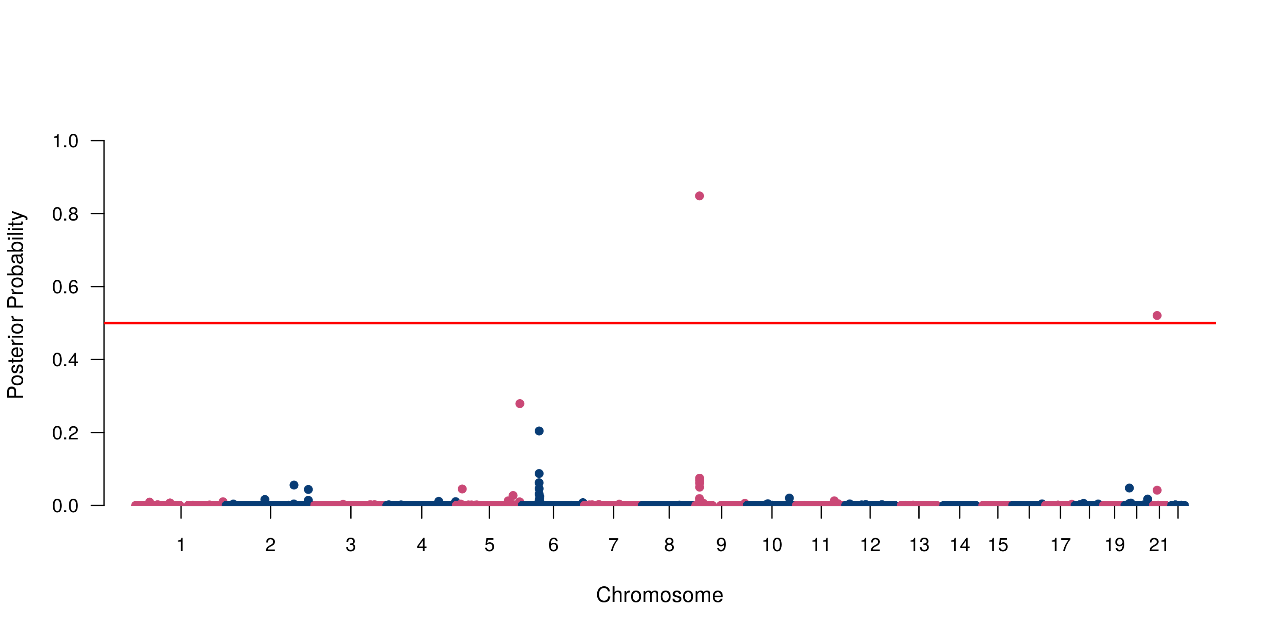


BMIX – SOUTHEAST ASIAN


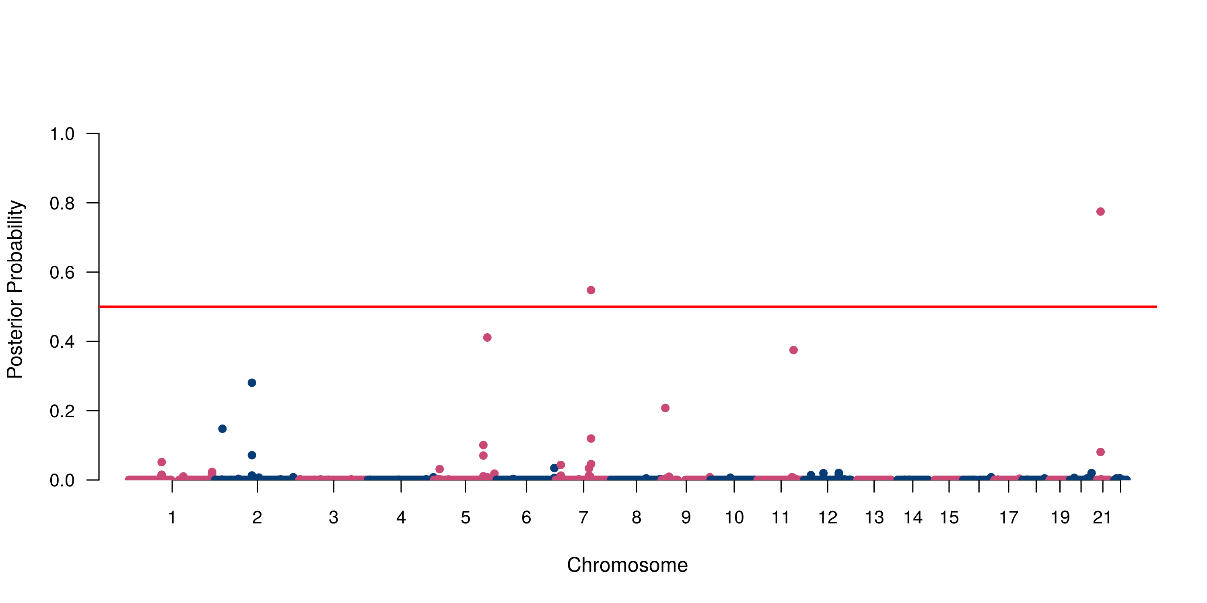


| Chr | SNP | BP | Allele | Association p-value | OR | BMIX posterior p-value in Northeast Asian ancestry | BMIX posterior p-value in Southeast Asian ancestry | Gene |
| --- | --- | --- | --- | --- | --- | --- | --- | --- |
| **RUN 2** | | | | | | | | |
| 22 | rs5761313 | 26313745 | T | 0.0001115 | 1.919 | 0.5009741 | 0.8918858 | *MYO18B* |
| 22 | rs2301504 | 26320015 | C | 0.0002039 | 1.835 |  | 0.5818690 | *MYO18B* |

Association


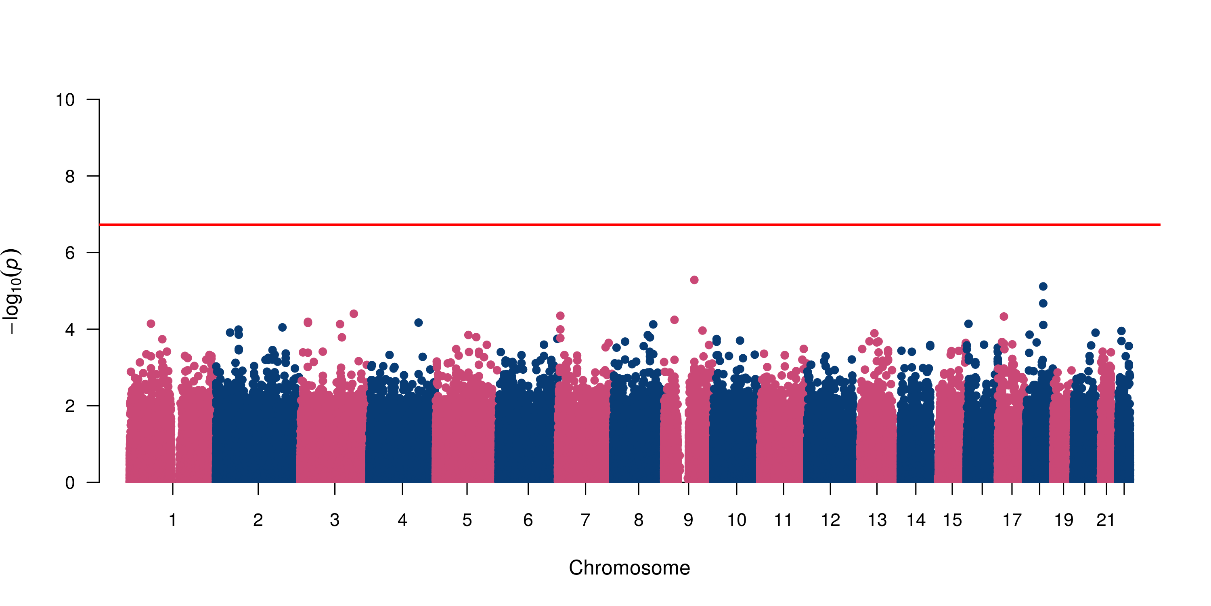


BMIX - NORTHEAST ASIAN


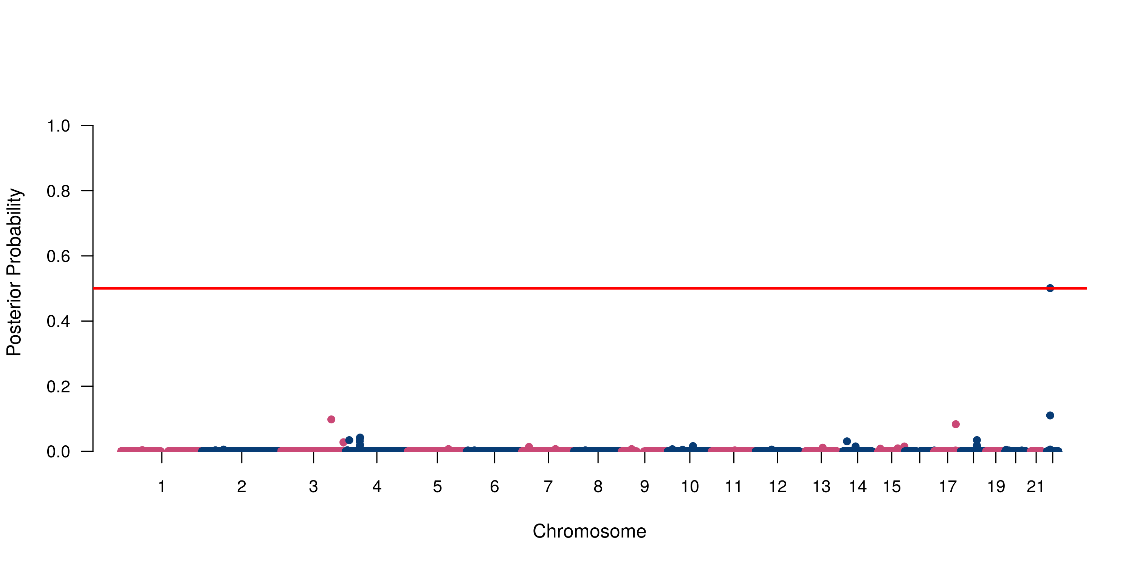


BMIX – SOUTHEAST ASIAN


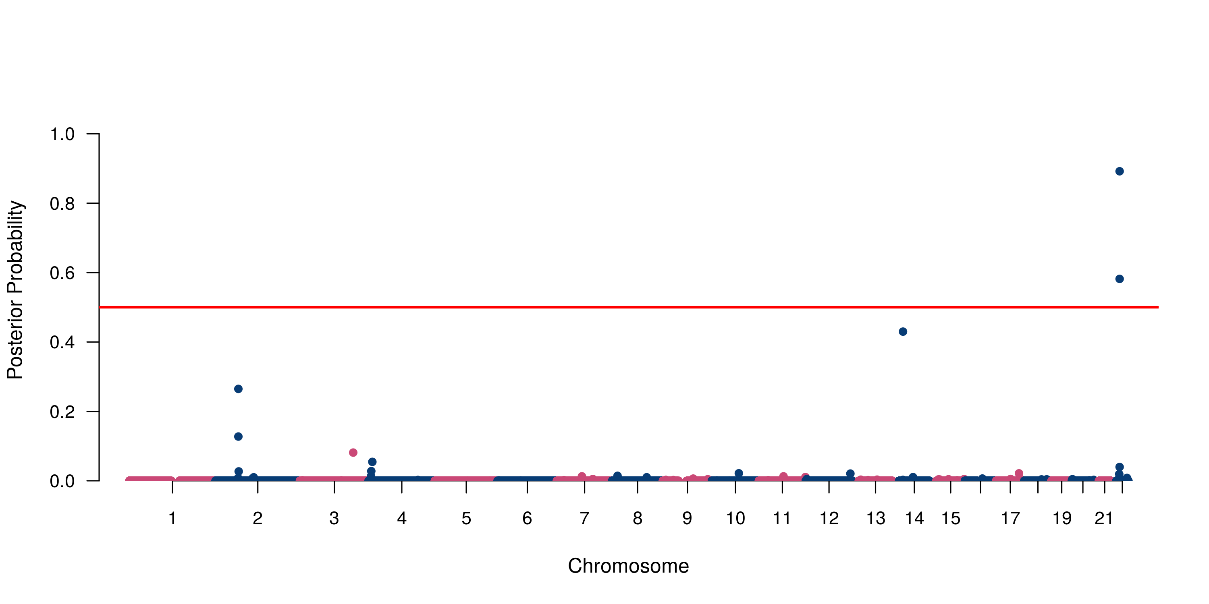


| Chr | SNP | BP | Allele | Association p-value | OR | BMIX posterior p-value in Northeast Asian ancestry | BMIX posterior p-value in Southeast Asian ancestry | Gene |
| --- | --- | --- | --- | --- | --- | --- | --- | --- |
| **RUN 3** | | | | | | | | |
| 6 | rs9496739 | 144020900 | A | 3.457e-05 | 0.551 | 0.5527984 |  | *PHACTR2* |
| 17 | rs12709500 | 26134974 | C | 1.326e-05 | 0.4801 |  | 0.7308197 | Intergenic |
| 17 | rs16949120 | 48610732 | A | 8.477e-05 | 2.491 | 0.9197636 |  | *EPN3/MYCBPAP* |

Association


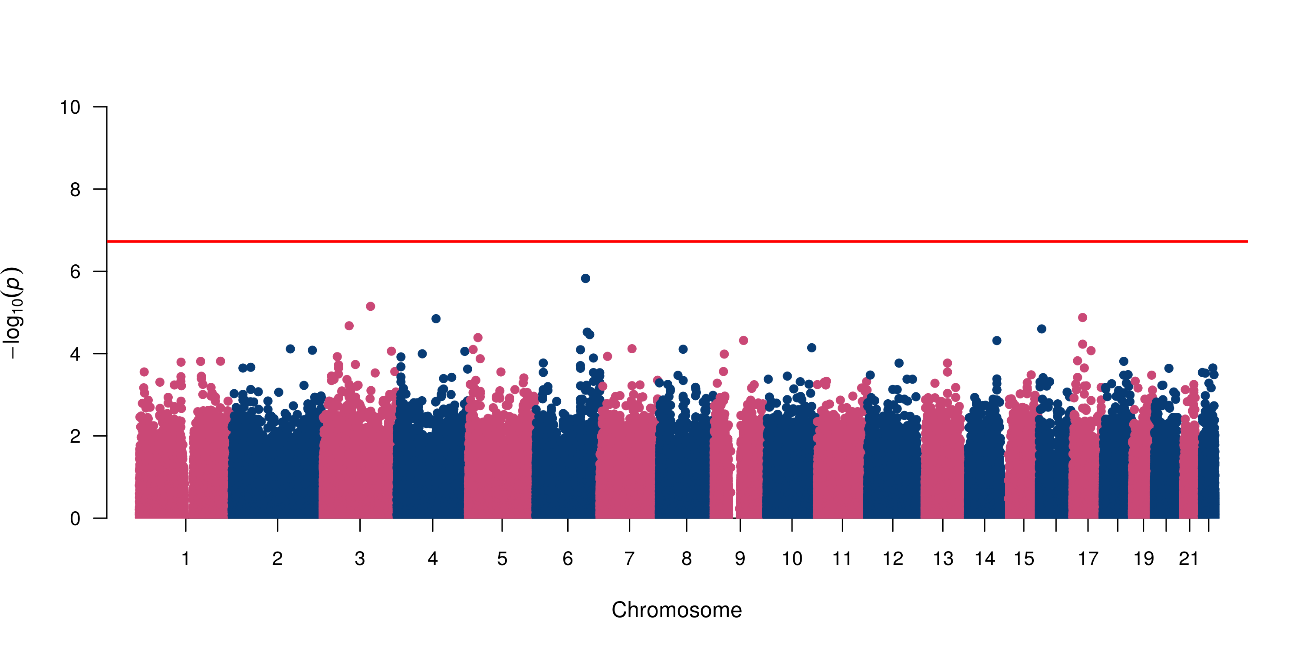


BMIX - NORTHEAST ASIAN


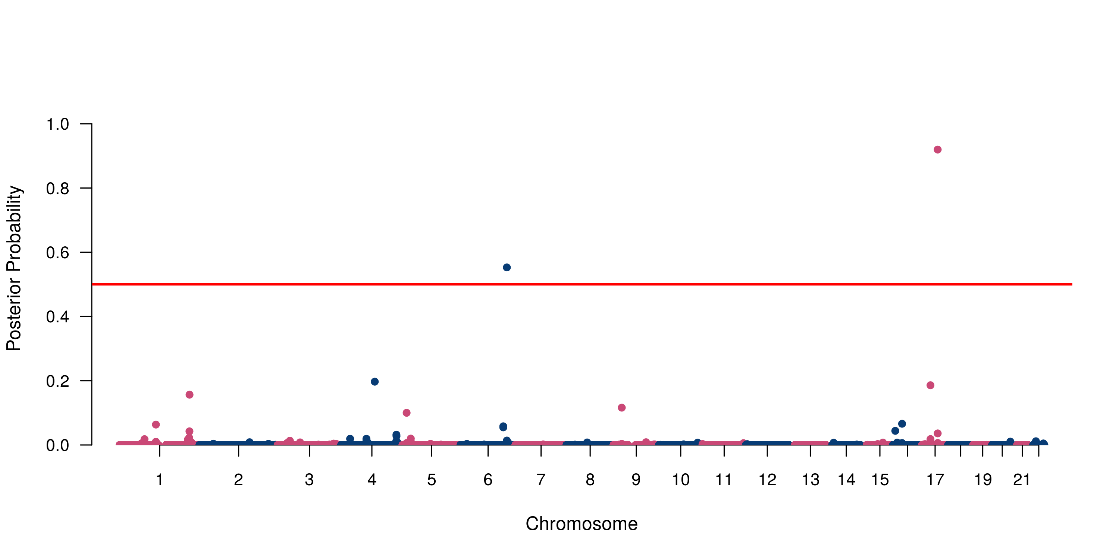


BMIX – SOUTHEAST ASIAN


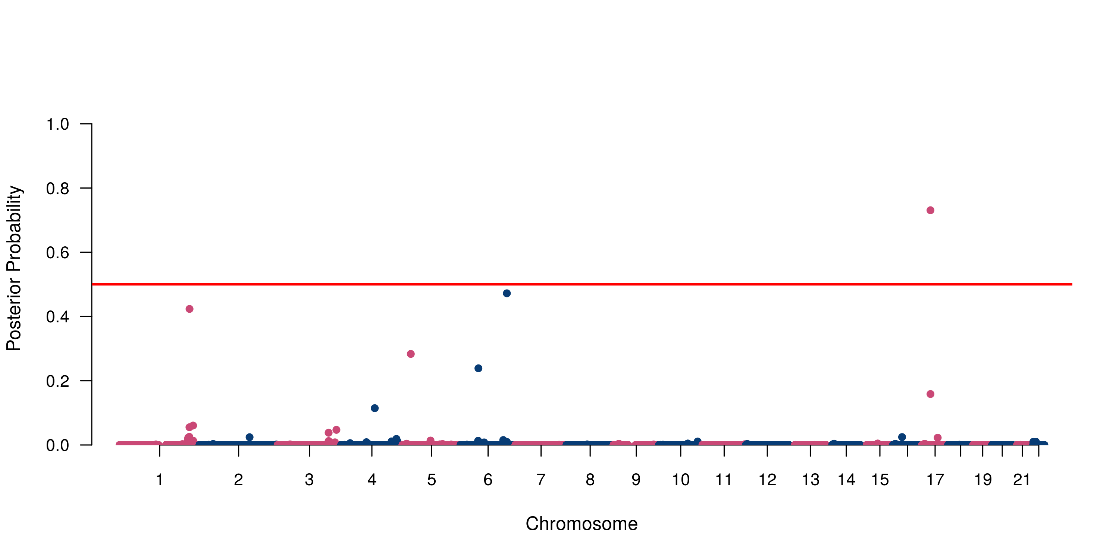


| Chr | SNP | BP | Allele | Association p-value | OR | BMIX posterior p-value in Northeast Asian ancestry | BMIX posterior p-value in Southeast Asian ancestry | Gene |
| --- | --- | --- | --- | --- | --- | --- | --- | --- |
| **RUN 4** | | | | | | | | |
| 1 | rs2268170 | 9315847 | T | 2.19e-05 | 2.182 | 0.8404511 |  | *H6PD* |
| 14 | rs1456988 | 98488007 | T | 0.0005011 | 1.631 |  | 0.6064477 | Intergenic |
| 15 | rs1553893 | 26114658 | C | 0.0003323 | 2.802 | 0.6392913 |  | *ATP10A* |

Association


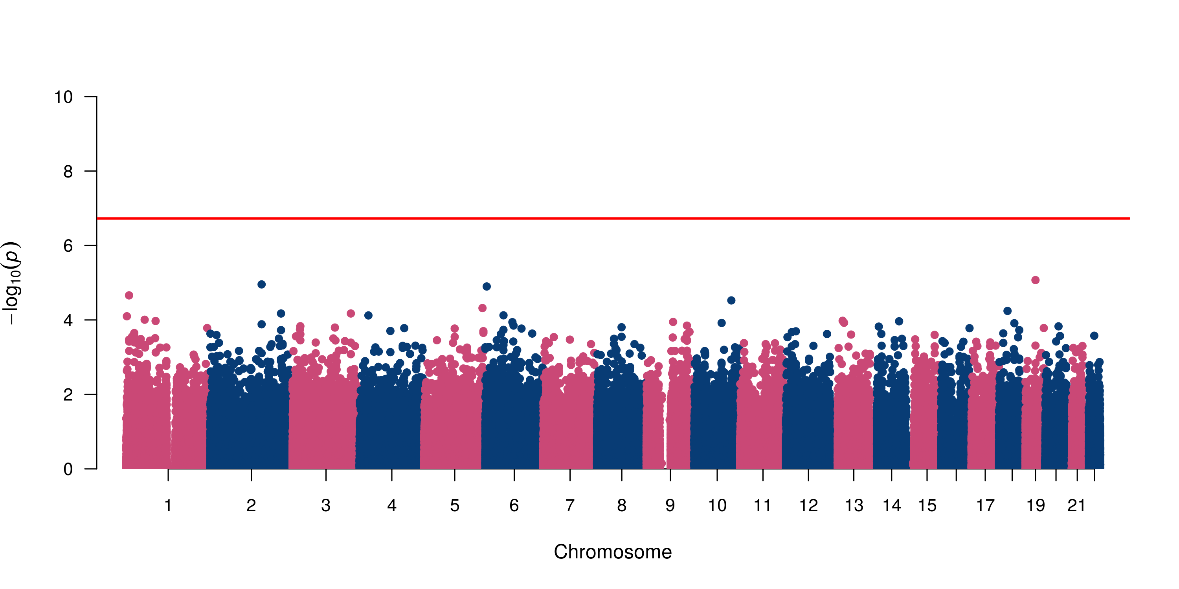


BMIX – NORTHEAST ASIAN


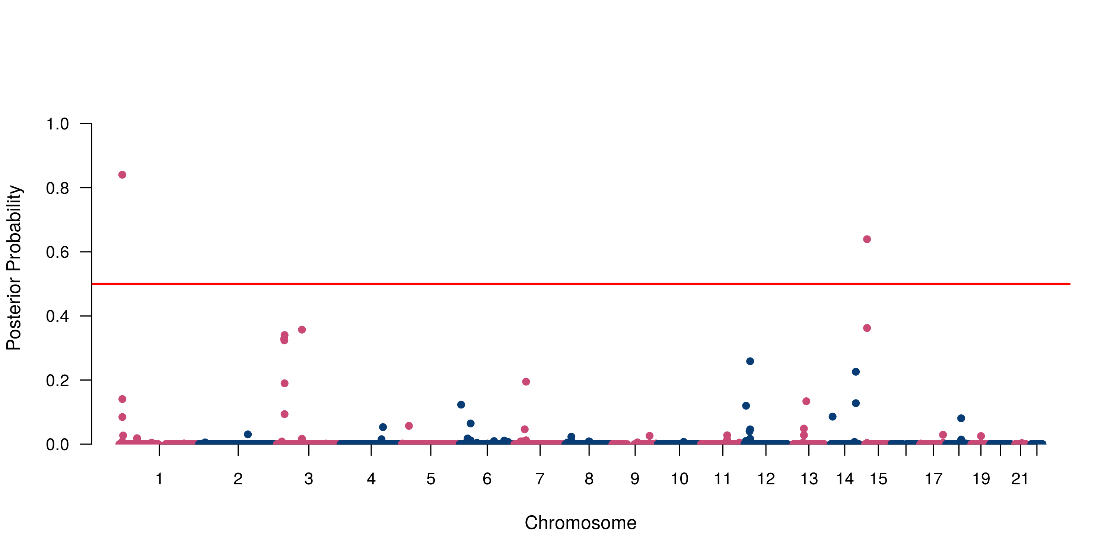


BMIX – SOUTHEAST ASIAN


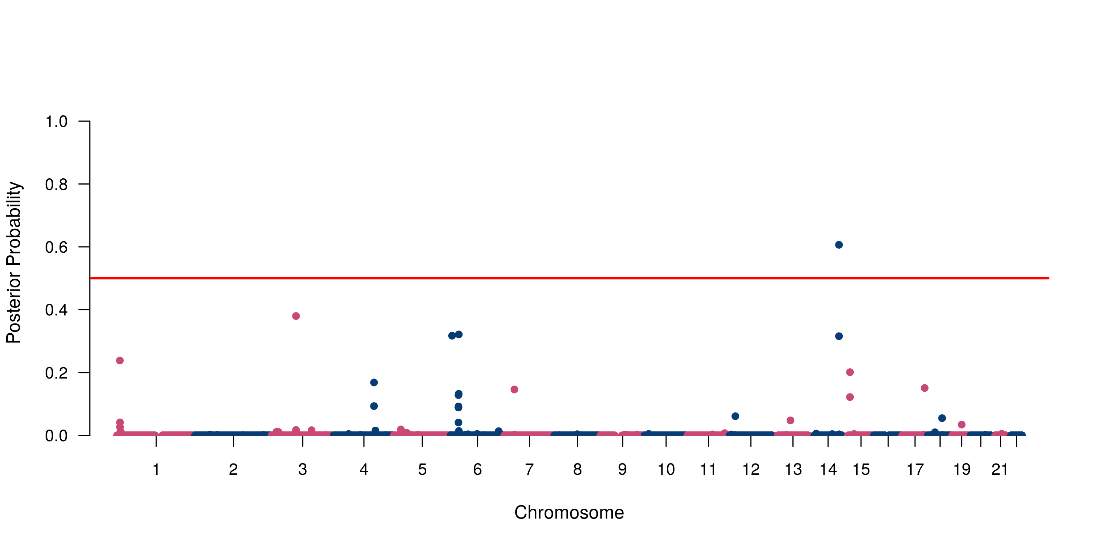


| Chr | SNP | BP | Allele | Association p-value | OR | BMIX posterior p-value in Northeast Asian ancestry | BMIX posterior p-value in Southeast Asian ancestry | Gene |
| --- | --- | --- | --- | --- | --- | --- | --- | --- |
| **RUN 5** | | | | | | | | |
| 2 | rs12990473 | 124454519 | A | 0.002542 | 0.652 | 0.6197150 |  | Intergenic |
| 8 | rs7002197 | 73123327 | G | 1.813e-05 | 1.978 |  | 0.7725738 | Intergenic |
| 9 | rs12683636 | 16862691 | C | 0.0002618 | 2.008 |  | 0.7176504 | *BNC2* |
| 11 | rs10838216 | 44038877 | C | 0.0005432 | 1.752 |  | 0.7115631 | Intergenic |
| 12 | rs7305703 | 38138381 | C | 0.0003085 | 2.607 |  | 0.5054825 | Intergenic |
| 18 | rs7226876 | 56532820 | T | 1.979e-05 | 2.123 | 0.8451823 |  | *ZNF532* |

Association


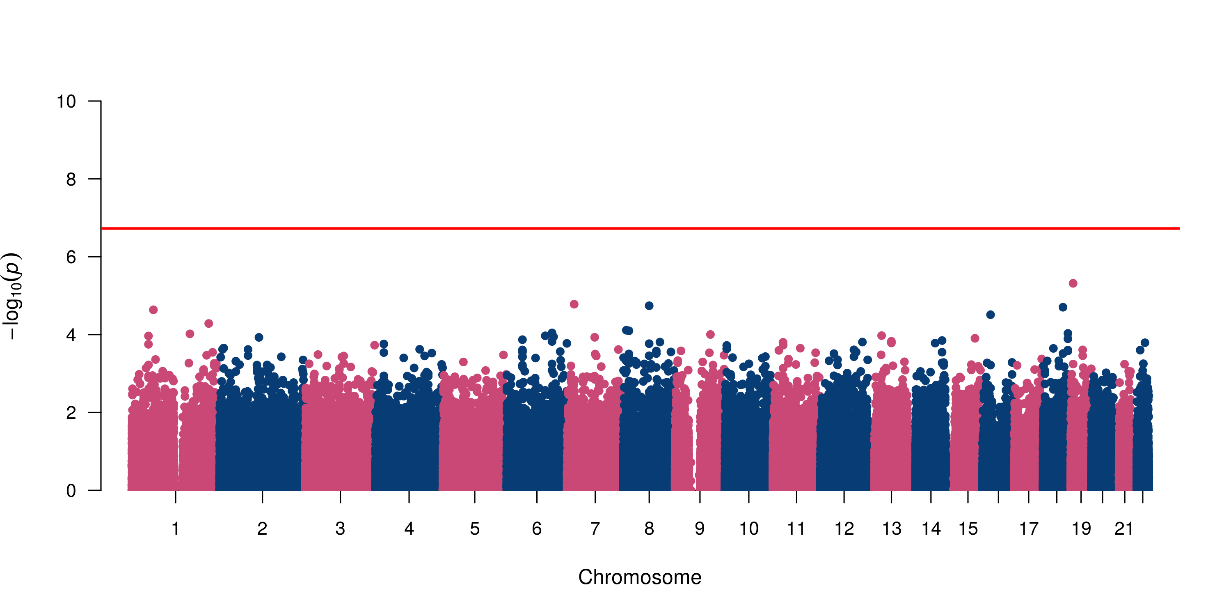


BMIX – NORTHEAST ASIAN


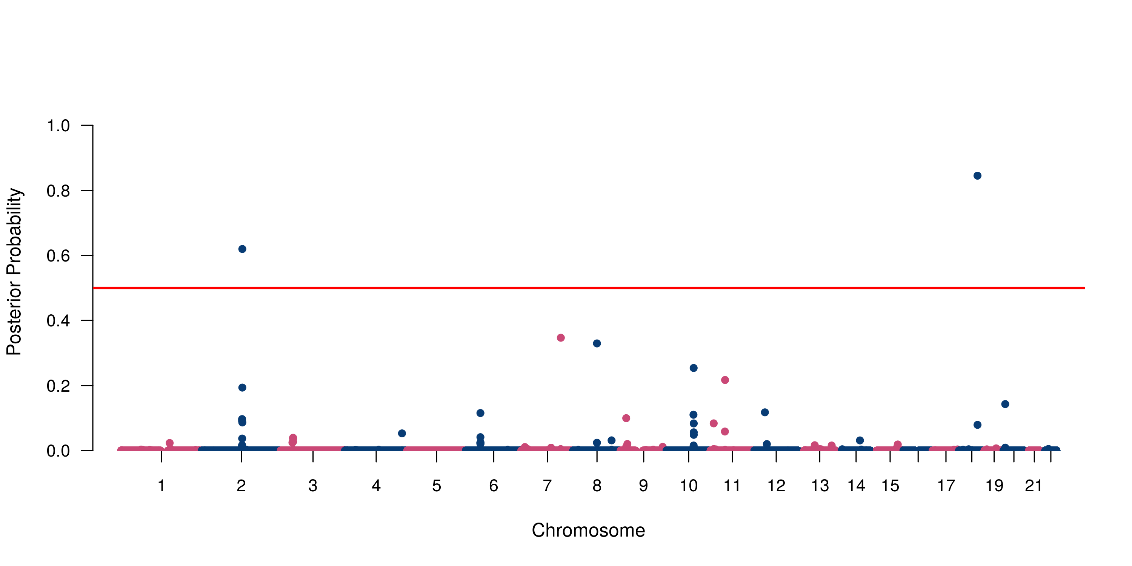


BMIX – SOUTHEAST ASIAN


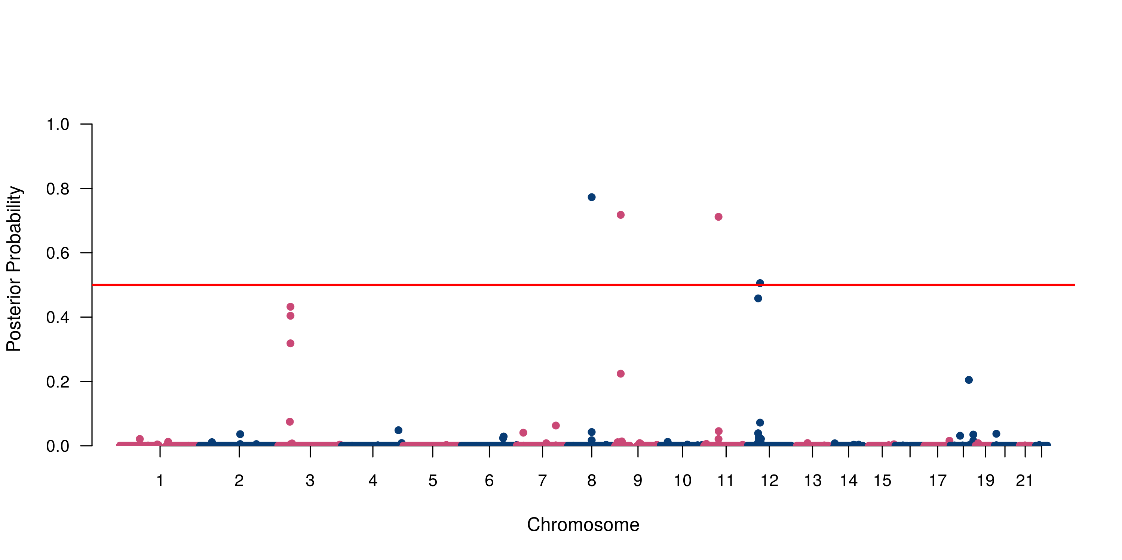


| Chr | SNP | BP | Allele | Association p-value | OR | BMIX posterior p-value in Northeast Asian ancestry | BMIX posterior p-value in Southeast Asian ancestry | Gene |
| --- | --- | --- | --- | --- | --- | --- | --- | --- |
| **RUN 6** | | | | | | | | |
| 2 | rs7602673 | 121853648 | C | 0.0004952 | 1.633 |  | 0.6372767 | Intergenic |
| 9 | rs2295870 | 5436461 | C | 0.0003478 | 0.5824 |  | 0.5744294 | *PLGRKT* |

Association


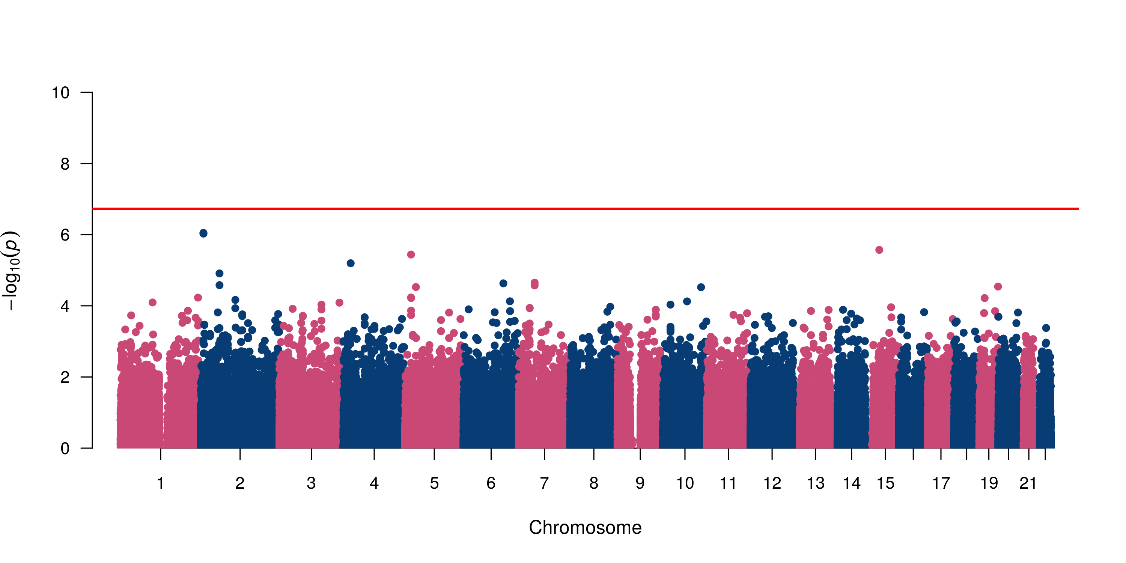


BMIX – NORTHEAST ASIAN


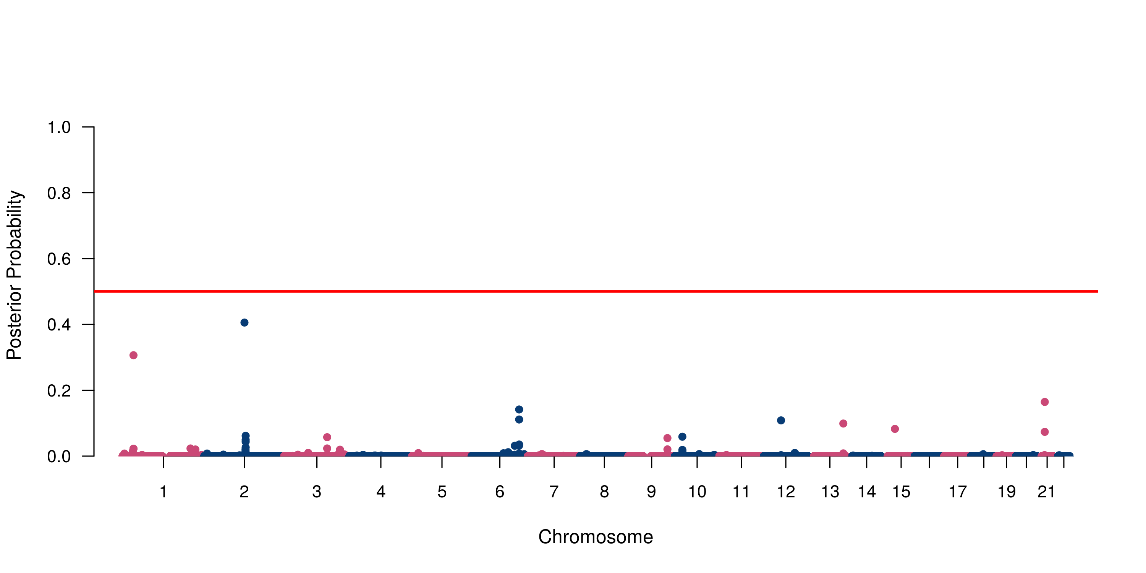


BMIX – SOUTHEAST ASIAN


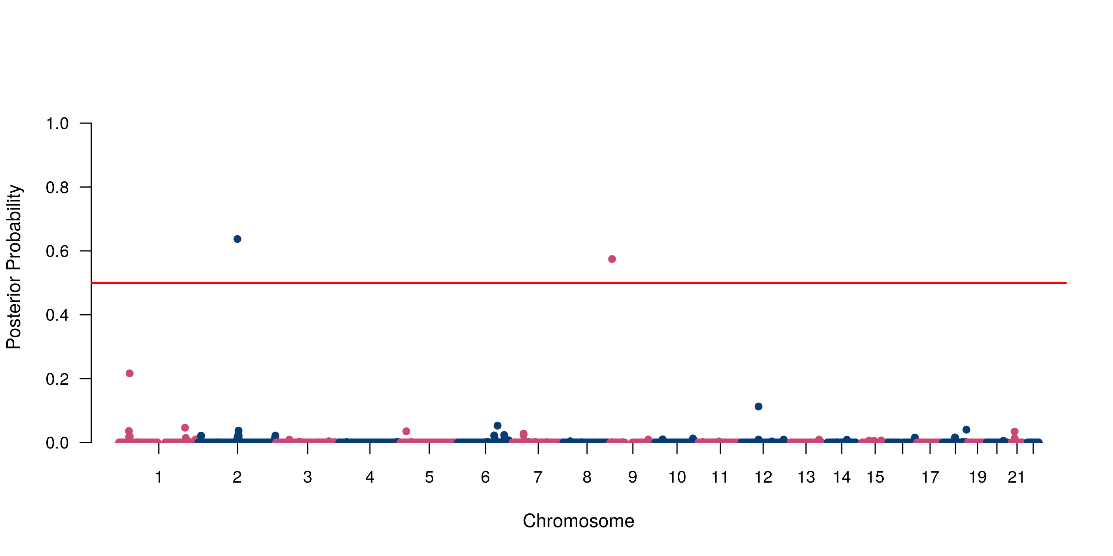


| Chr | SNP | BP | Allele | Association p-value | OR | BMIX posterior p-value in Northeast Asian ancestry | BMIX posterior p-value in Southeast Asian ancestry | Gene |
| --- | --- | --- | --- | --- | --- | --- | --- | --- |
| **RUN 7** | | | | | | | | |
| 2 | rs4851346 | 101223405 | T | 0.0001164 | 1.736 | 0.5367466 |  | Intergenic |
| 2 | rs12988520 | 234607394 | C | 1.53e-05 | 1.870 | 0.7438419 |  | *UGT1A6/7/8/9/10* |
| 3 | rs805478 | 70427067 | T | 2.257e-05 | 2.365 | 0.8182922 | 0.8677060 | Intergenic |
| 6 | rs9385270 | 123092085 | T | 1.767e-06 | 1.983 |  | 0.5599361 | Intergenic |
| 7 | rs7811417 | 21534152 | T | 0.0001379 | 0.580 |  | 0.8249133 | *SP4* |

Association


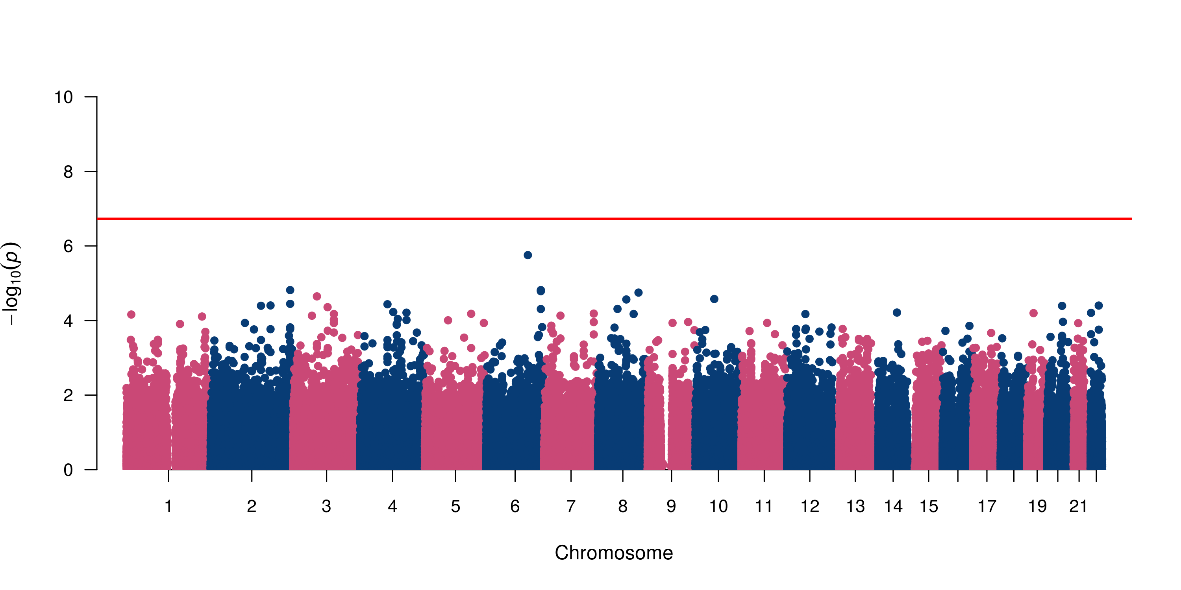


BMIX – NORTHEAST ASIAN


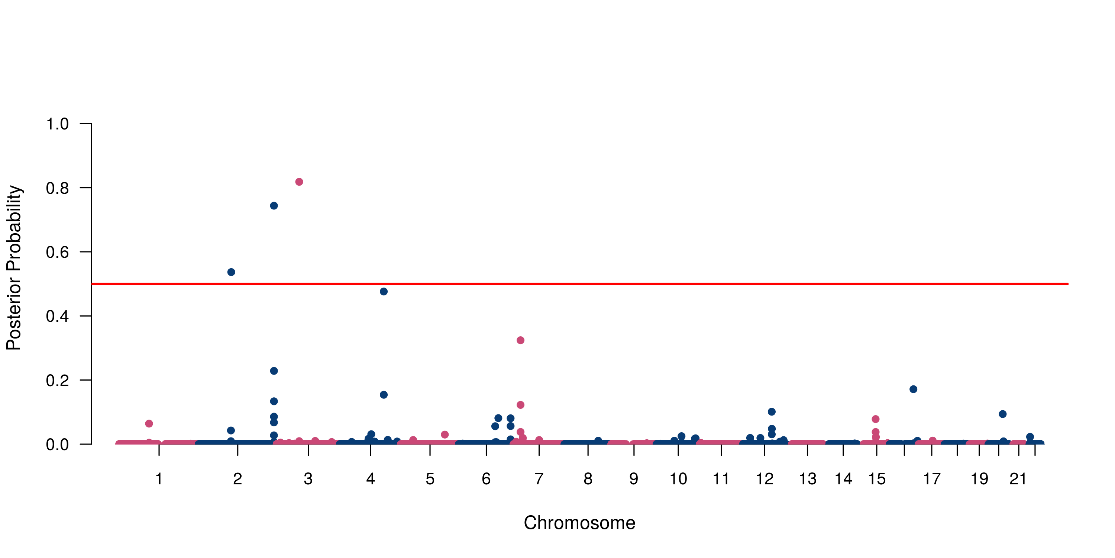


BMIX – SOUTHEAST ASIAN


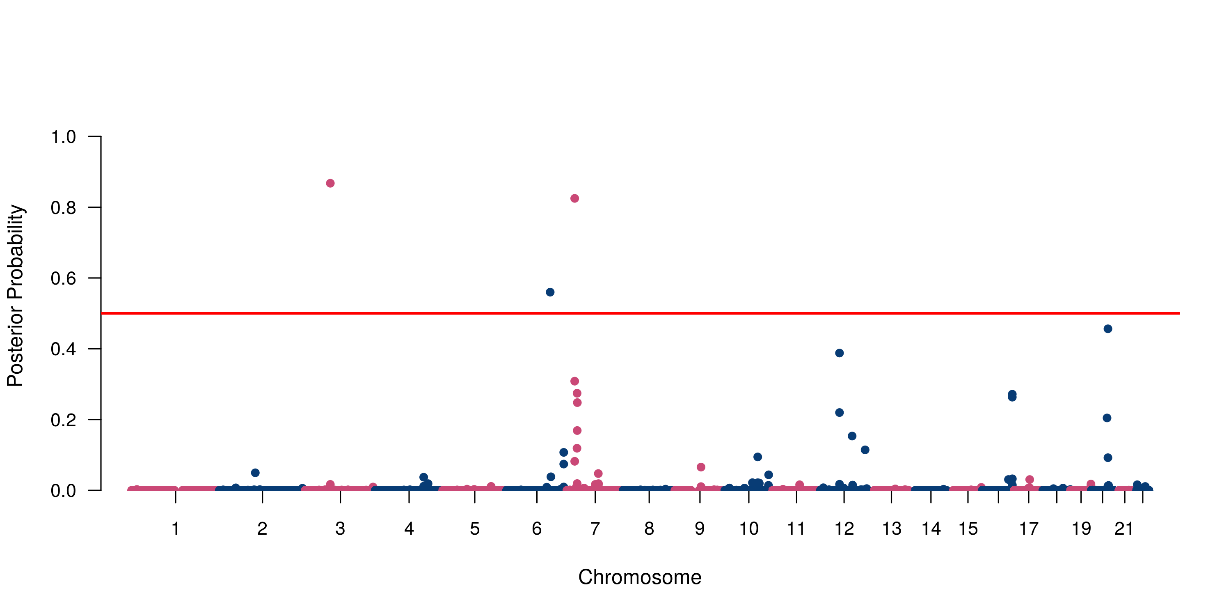


| Chr | SNP | BP | Allele | Association p-value | OR | BMIX posterior p-value in Northeast Asian ancestry | BMIX posterior p-value in Southeast Asian ancestry | Gene |
| --- | --- | --- | --- | --- | --- | --- | --- | --- |
| **RUN 8** | | | | | | | | |
| 2 | rs4246580 | 50897456 | G | 0.0001965 | 1.757 | 0.5729017 |  | *NRXN1* |
| 5 | rs1450624 | 165956290 | G | 0.0008588 | 1.769 |  | 0.6864852 | Intergenic |
| 19 | rs2285963 | 5591735 | A | 2.499e-05 | 2.509 |  | 0.7265687 | *SAFB2* |

Association


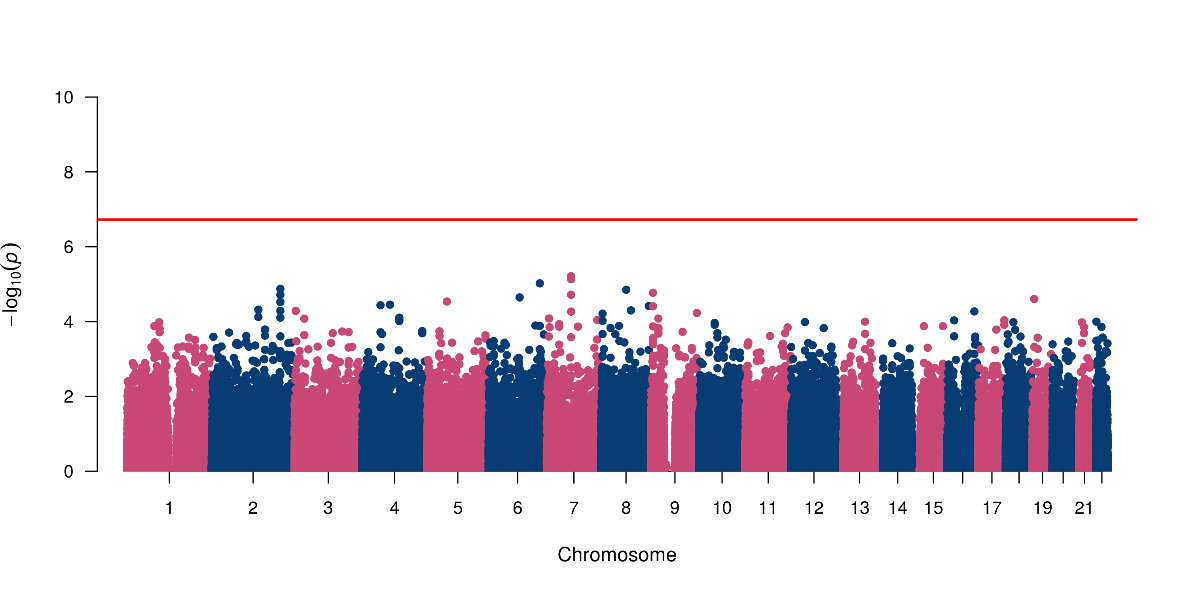


BMIX – NORTHEAST ASIAN


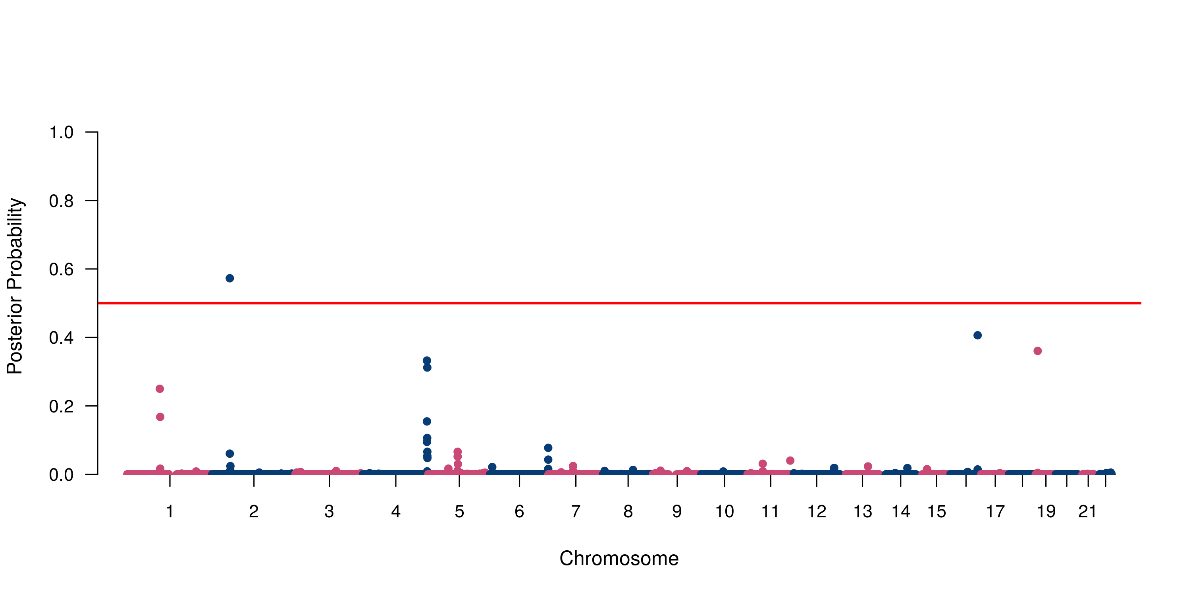


BMIX – SOUTHEAST ASIAN


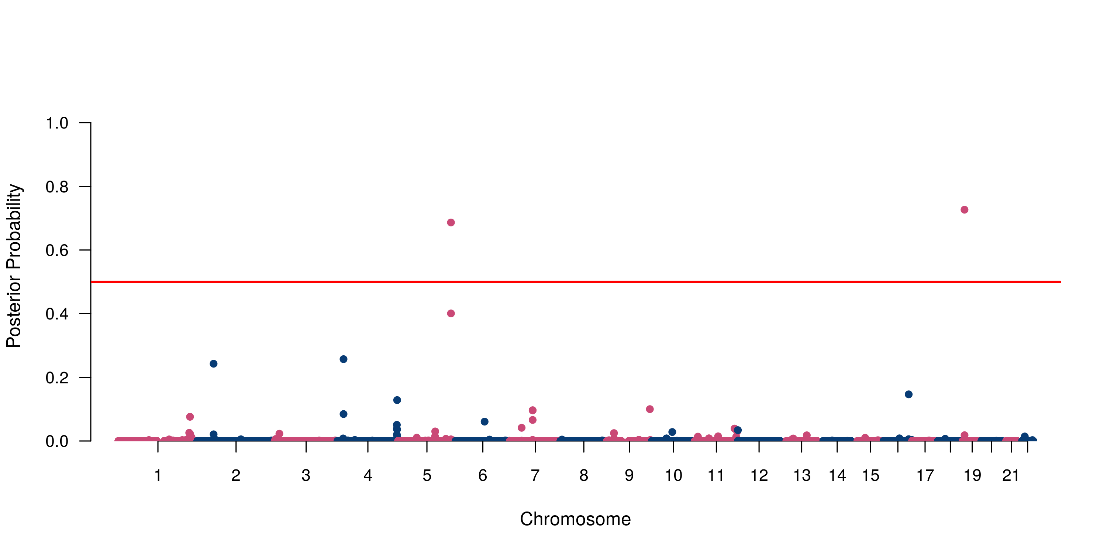


| Chr | SNP | BP | Allele | Association p-value | OR | BMIX posterior p-value in Northeast Asian ancestry | | BMIX posterior p-value in Southeast Asian ancestry | Gene |
| --- | --- | --- | --- | --- | --- | --- | --- | --- | --- |
| **RUN 9** | | | | | | | | | |
| 10 | rs2907567 | 117618612 | T | 0.0003373 | 0.489 | 0.7767757 |  | | *ATRNL1* |
| 12 | rs12314724 | 130274359 | C | 0.0001051 | 0.452 |  | 0.6794178 | | *TMEM132D* |
| 17 | rs11869840 | 44996100 | A | 1.194e-06 | 1.985 |  | 0.8533168 | | *GOSR2* |
| 17 | rs1662576 | 44996245 | T | 3.702e-06 | 1.923 |  | 0.5918913 | | *GOSR2* |

Association


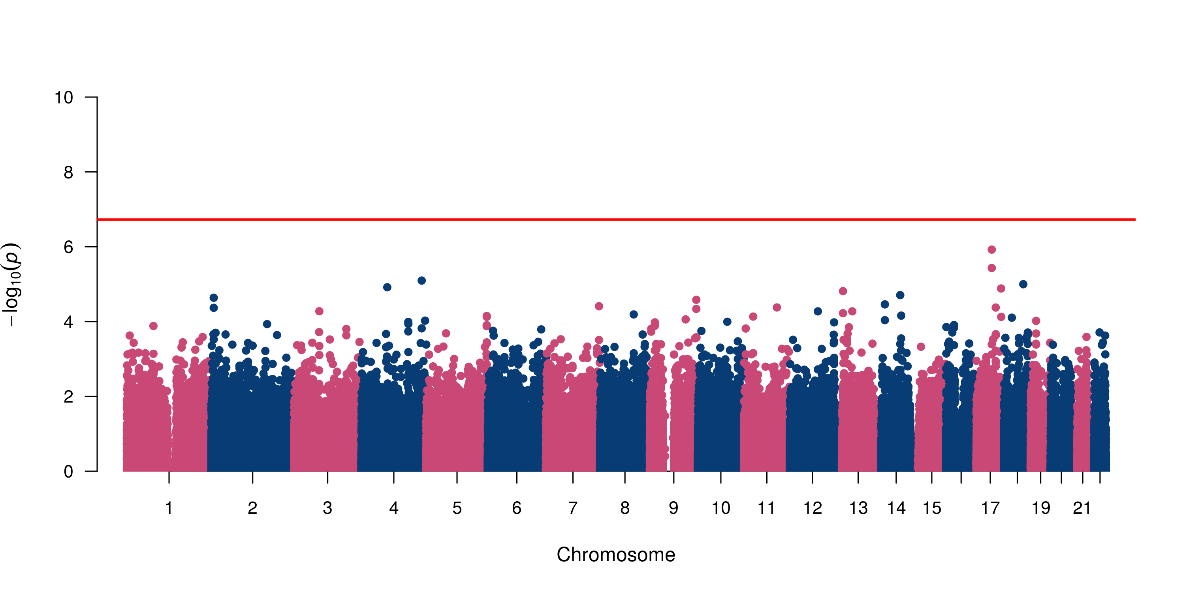


BMIX – NORTHEAST ASIAN


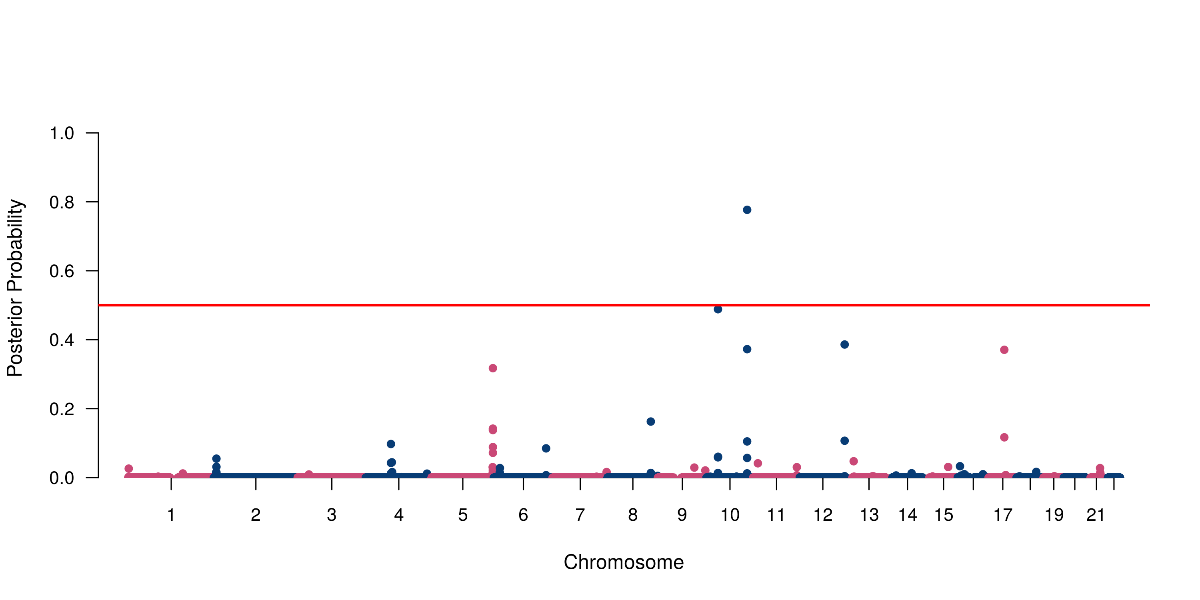


BMIX – SOUTHEAST ASIAN


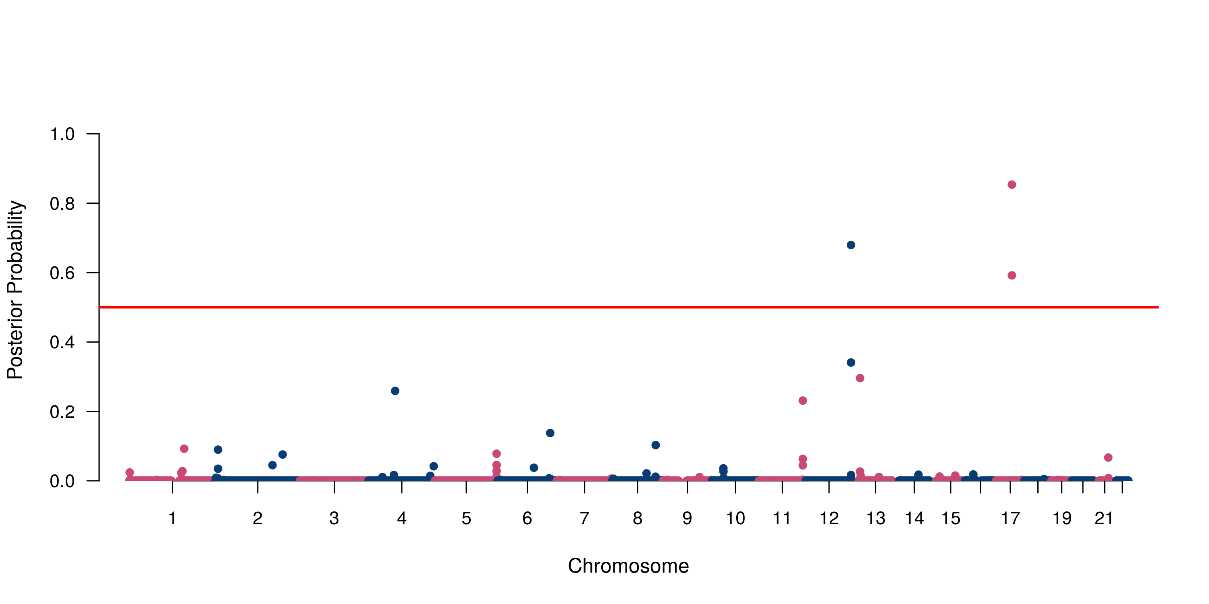


| Chr | SNP | BP | Allele | Association p-value | OR | BMIX posterior p-value in Northeast Asian ancestry | BMIX posterior p-value in Southeast Asian ancestry | Gene |
| --- | --- | --- | --- | --- | --- | --- | --- | --- |
| **RUN 10** | | | | | | | | |
| 7 | rs9656687 | 53635159 | A | 8.489e-05 | 0.495 |  | 0.7990117 | Intergenic |
| 7 | rs7803594 | 53654002 | T | 0.0001859 | 0.588 |  | 0.5609658 | Intergenic |
| 7 | rs672416 | 105376750 | T | 2.829e-06 | 2.005 |  | 0.6579472 | *ATXN7L1* |
| 7 | rs577004 | 105383875 | C | 3.066e-06 | 2.018 |  | 0.5470686 | *ATXN7L1* |

Association


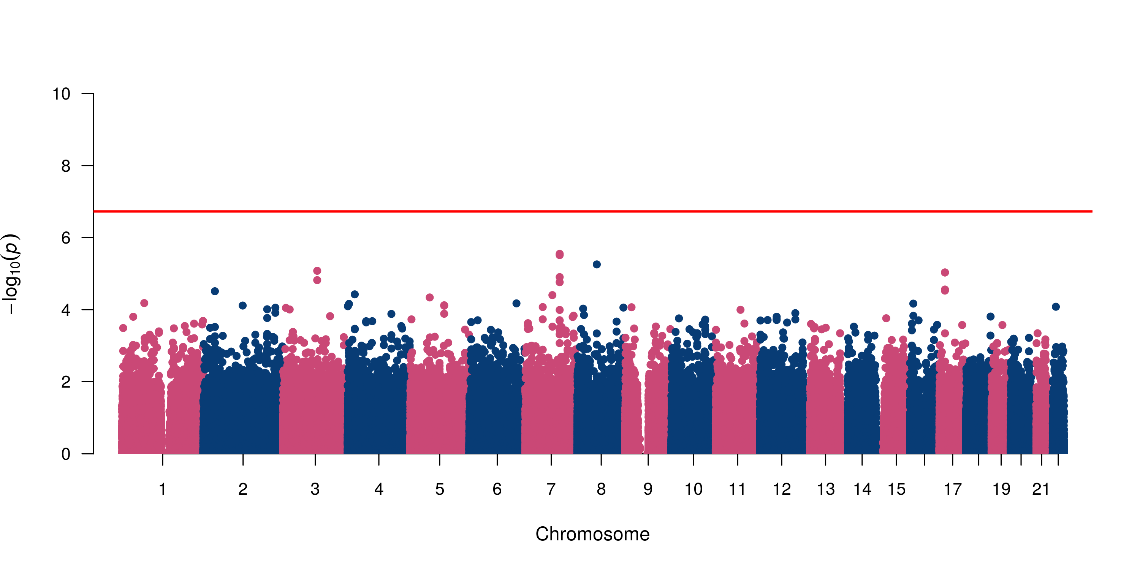


BMIX - NORTHEAST ASIAN


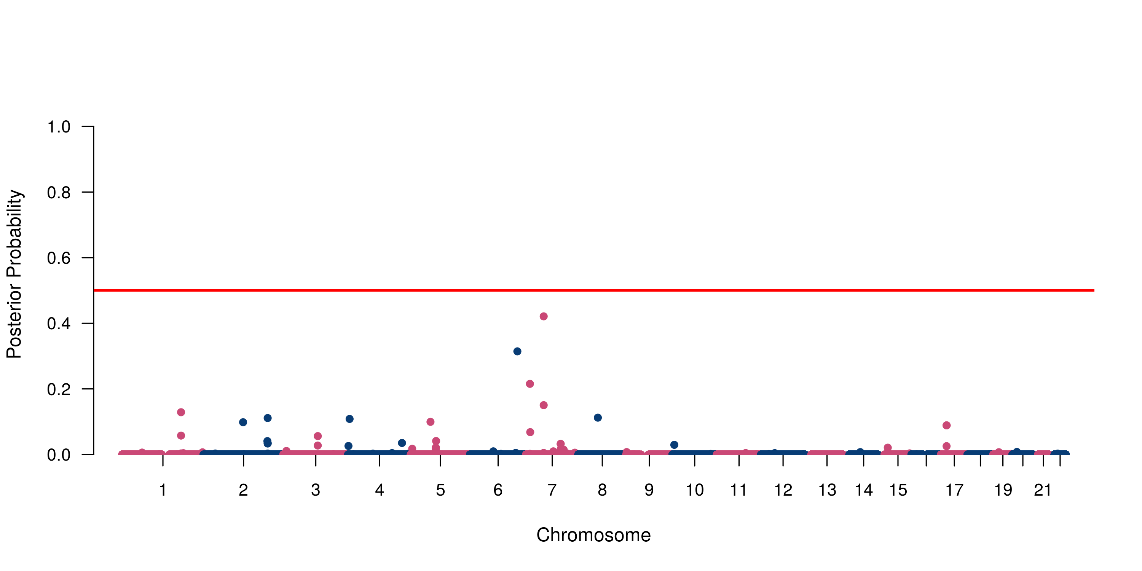


BMIX – SOUTHEAST ASIAN


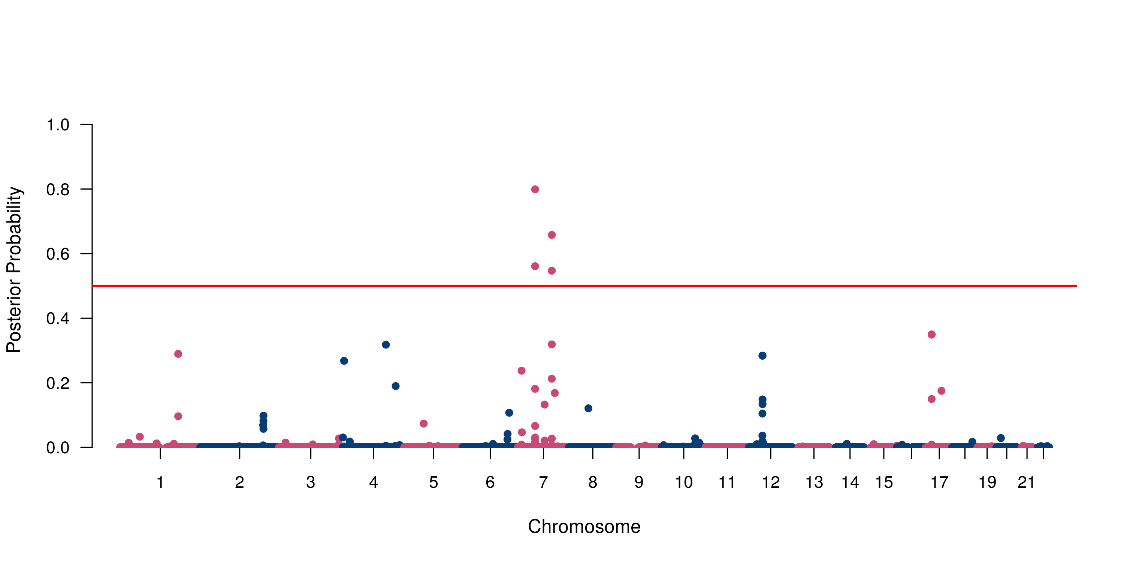

Supplement: S1 Text — (DOCX) [file pntd.0006202.s024.docx]
